# Behind the screen: drug discovery using the big data of phenotypic analysis

**DOI:** 10.3389/feduc.2024.1342378

**Published:** 2024-02-14

**Authors:** Merrill M. Froney, Michael B. Jarstfer, Samantha G. Pattenden, Amanda C. Solem, Olubunmi O. Aina, Melissa R. Eslinger, Aeisha Thomas, Courtney M. Alexander

**Affiliations:** 1 Department of Chemical Biology and Medicinal Chemistry, UNC Eshelman School of Pharmacy, University of North Carolina at Chapel Hill, Chapel Hill, NC, United States; 2 Department of Biology, Hastings College, Hastings, NE, United States; 3 Department of Biology, Allen University, Columbia, SC, United States; 4 Department of Chemistry and Life Science, United States Military Academy, West Point, NY, United States; 5 Department of Biological and Health Sciences, Crown College, St. Bonifacius, MN, United States; 6 Department of Biology, University of North Carolina at Pembroke, Pembroke, NC, United States

**Keywords:** high throughput screening, drug discovery, active learning, education, case study, big data

## Abstract

Technological advances in drug discovery are exciting to students, but it is challenging for faculty to maintain the pace with these developments, particularly within undergraduate courses. In recent years, a High-throughput Discovery Science and Inquiry-based Case Studies for Today’s Students (HITS) Research Coordination Network has been assembled to address the mechanism of how faculty can, on-pace, introduce these advancements. As a part of HITS, our team has developed “Behind the Screen: Drug Discovery using the Big Data of Phenotypic Analysis” to introduce students and faculty to phenotypic screening as a tool to identify inhibitors of diseases that do not have known cellular targets. This case guides faculty and students though current screening methods using statistics and can be applied at undergraduate and graduate levels. Tested across 70 students at three universities and a variety of courses, our case utilizes datasets modeled on a real phenotypic screening method as an accessible way to teach students about current methods in drug discovery. Students will learn how to identify hit compounds from a dataset they have analyzed and understand the biological significance of the results they generate. They are guided through practical statistical procedures, like those of researchers engaging in a novel drug discovery strategy. Student survey data demonstrated that the case was successful in improving student attitudes in their ability to discuss key topics, with both undergraduate and graduate students having a significant increase in confidence. Together, we present a case that uses big data to examine the utility of a novel phenotypic screening strategy, a pedagogical tool that can be customized for a wide variety of courses.

## Introduction

Constant innovation in drug discovery makes it difficult for undergraduate courses to access up-to-date technology for teaching current methods in pharmaceutical research. Exposing students to large data sets collected or modeled by data generated in real laboratories can help increase engagement (Freeman et al., 2014; Kontra et al., 2015) and allows universities to provide students with hands-on activities without having to budget for expensive lab equipment. This type of pedagogical tool would be especially helpful for teaching current methods in pharmaceutical science as lab equipment for these methods are expensive, hard to maintain, and are sometimes not very accessible due to privacy within industry and academia.

With high throughput screening and big data analysis becoming more vital in scientific fields, it is important for students to be trained in these methods to make them more prepared for future careers in STEM (Miller, 2014; Stephens et al., 2015; Barone et al., 2017; Howe et al., 2017; Williams and Teal, 2017). A High-throughput Discovery Science and Inquiry-based Case Studies for Today’s Students (HITS) Research Coordination Network has been assembled to address how faculty can introduce advancements in STEM fields. The HITS network was motivated by the slow progress undergraduate programs had made toward updating curricula to more modern quantitative standards (Robertson et al., 2021). The goal of HITS was to develop innovative curriculum materials in the form of case-based studies that involve hands-on activities with large high throughput datasets. The HITS initiative has built an interactive network that has successfully circulated high throughput case-based datasets across the country while also generating tools to help instructors develop their own case-based lesson plans (Bixler et al., 2021; Robertson et al., 2021). High throughput cases developed by the HITS network directly address common barriers to incorporating big data into curricula by using publicly available datasets, well detailed teaching notes, and highly adaptable cases (Williams et al., 2019). Case studies are a great way for students to learn high throughput methodology in tandem with high throughput quantitative skills (Samsa et al., 2021). Problem-based learning tools, such as case studies, urge students to solve real world problems which improves student motivation to learn and understand key topics (Gallagher et al., 1995; Lombardi and Oblinger, 2007). Case studies are interactive and faculty that have implemented case studies in their curriculum have observed an increase in student critical thinking and understanding of scientific concepts (Yadav et al., 2007).

High throughput screening is necessary for drug development with screens developed and optimized for a large variety of target pathways. Providing students with case studies and real-world datasets can teach students how to analyze high throughput screening data in addition to interactive teaching of current methods in drug discovery. In drug discovery there are two types of screens: target-based and phenotypic-based (Swinney, 2013). Target-based screens are used when a cellular target is known to be involved in disease progression and are based on change in activity of a specific protein with a known role in the cellular pathway of interest (Croston, 2017). Target-based screens are ideal for diseases with known cellular targets but are not applicable for drug discovery for diseases with no known cellular targets. Phenotypic screening is based on a cellular biomarker and is often target agnostic. Phenotypic screens are useful for discovering new therapeutic targets but are harder to optimize for high throughput use and may need more customized statistical metrics compared to target-based screens (Moffat et al., 2017). Phenotypic screens have been successful in drug discovery campaigns for a variety of diseases such as bacterial and parasitic infection (Battah et al., 2019; Saccoccia et al., 2020). “Behind the Screen: Drug Discovery using the Big Data of Phenotypic Analysis” describes the development and use of a high throughput screen for detecting compounds that interfere with a cancer-specific pathway from the perspective of a graduate student. Students learn the difference between target-based and phenotypic-based screens (Swinney, 2013) and how experimental design and statistical analysis differs depending on assay readout (Markossian et al., 2004; Zhang, 2011). Target-based and phenotypic screening methods are very different, especially in the type of samples used in screening and assay readout (Strovel et al., 2016) (see supplemental teaching notes). These differences lead to variation in how datasets from each type of screen are analyzed. Customizing statistical analysis to best match the scientific protocol is very important and must be done without compromising a researcher’s ethical responsibility in data reporting. It has been reported that a large percentage of published research articles do not report statistical analysis properly or responsibly (Chiu et al., 2017; Diong et al., 2018). Incorrect data analysis and interpretation can have drastic effects on the development of future studies in all fields by inaccurately informing researchers. It is important for authors to understand how to properly choose statistical methods and report their results responsibly. In high throughput screening campaigns, methods of statistical analysis should be chosen based on experimental design and parameters of the data rather than which method gives desired results (Markossian et al., 2004; Zhang, 2011; Lindner et al., 2018). This case study introduces students to drug discovery screening techniques while also prompting them to think critically about the ethics of statistical analysis and data reporting.

This case is customizable, making it applicable to a wide variety of curriculums. The case discusses cancer biology (Griffith et al., 1999; Henson et al., 2009; Cesare and Reddel, 2010), high throughput screening, statistics, and ethics in science (see supplemental teaching notes). Any combination of these topics can be emphasized for a particular course. To demonstrate the adaptability of the case, we implemented it in 4 undergraduate courses (BIOL 459: Molecular Biology, SCI 458: Scientific Research and Analysis, BIO 4610: Animal Physiology, and PHRS 500: Innovations and Transformations in Pharmacy and Pharmaceutical Sciences) and a graduate level course (PHRS 802: Introduction to Drug Development). Each implementation was catered toward course curriculum while also meeting the case learning objectives. We found that our implementations in undergraduate and graduate courses met the learning objectives and improved student comfort in discussing the case material.

### Pedagogical framework(s)

It is hard to give students hands-on experience with experimental methods in high throughput screening as the equipment needed to run experiments is costly, hard to maintain, and often hard to access. Case studies can be a valuable interactive tool for teaching topics involving high throughput screening and statistical analysis of large datasets (Mahdi et al., 2020). Here, we describe the implementations of “Behind the Screen: Drug Discovery using the Big Data of Phenotypic Analysis” in 4 undergraduate courses and 1 graduate course. The case study was taught to over 70 students across 3 universities in the United States and students were surveyed to assess the case’s ability to meet the learning objectives.

The lesson plan included a pre-class reading assignment and question set, an in-class lecture and data analysis activity, and students were sent home with a post-class homework assignment involving a data analysis activity and a question set. Students who consented to being evaluated were given paper surveys at the beginning and end of the in-class session to measure improvement of student understanding after the in-class portion of the lesson. The pre-class reading, teaching notes, in-class activity dataset, step-by-step instructions for data analysis, and homework dataset and questions are provided (Supplementary material).

## Methods: learning environment; learning objectives; pedagogical format

### Learning environment

#### PHRS 802 graduate level drug development and professional skills development

PHRS 802 was an introductory course for first year graduate students in the Pharmaceutical Sciences PhD program at the University of North Carolina at Chapel Hill. All 19 students in this course had at least a bachelor’s degree and the student age range was 22–35. The class met once a week for a 60 min in-person class session. One of the main purposes of this course was to expose students to methods commonly used in each stage of drug development (Sun et al., 2022). Since “Behind the Screen” describes high throughput screen development in the context of drug discovery, this case fit well into the course curriculum. For PHRS 802, we emphasized the case themes in high throughput drug discovery methods and customization of statistical analysis for phenotypic screening. The implementation of “Behind the Screen” was done in one 60-min class session of PHRS 802. The session included a 30-min lecture and a 30-min in-class activity (supplemental in class dataset). The in-class activity was done together as a whole class.

#### PHRS 500 innovations and transformations in pharmacy and pharmaceutical sciences

PHRS 500 was a summer course held at the University of North Carolina at Chapel Hill for undergraduate students interested in pursuing careers in pharmaceutical science. A majority of the 15 students in this course were visiting from out of the country and the age range of the class was 18–25. The goal of this course was to expose students to methods commonly used in each stage of drug development as well as give students an idea of what a graduate career in pharmaceutical sciences looks like. “Behind the Screen” fit very nicely into the PHRS 500 curriculum as it describes high throughput screen development in the context of drug discovery. For PHRS 500, we highlighted high throughput drug discovery methods and customization of statistical analysis for phenotypic screening. This case was especially applicable to PHRS 500 because it is written from the perspective of a graduate student. Since the participants in this course were interested in attending graduate school, this narrative gave them some insight into what graduate education might look like. The implementation of “Behind the Screen” was done in one 90-min class session of PHRS 500. The lecture took 30 min, leaving 60 min for the in-class activity. The in-class activity was done together as a whole class.

### SCI 458 scientific research and analysis

Scientific Research and Analysis SCI 458 is an upper-level class for undergraduates at Crown College. There were 6 students in the class and the prerequisites were Applied Statistics and at least one science course. This case study was relevant to this course since it exposed students to data analysis methods and decision-making. While the biology content was less relevant, students from a range of majors can understand the drug discovery process more broadly and appreciate the value. The pre-work (supplemental case study for students) was given and then the slides presented in class as the instructor walked through the case study with the students over about 3 50-min class periods. This implementation was the pilot run of the case study, and some changes were made which were then used in the remaining classes. Since changes were made to the lesson materials after this implementation, the data collected from this course was not included in the data analysis for this study.

### Bio 4610 animal physiology

Animal Physiology BIO 4610 is an upper-level class for undergraduate students at the University of North Carolina at Pembroke. The course functions as an advanced physiology course and is required for biomedical majors. There were 24 students in the class and the prerequisites were Anatomy and Physiology I and II. The class meets three times a week for 50 min for lecture and once a week for 90 min for lab. We used the case in our unit on data analysis and did not emphasize the cancer biology aspect. This case was especially relevant for the course, as most of the students were seniors applying to graduate school or medical school. The implementation of “Behind the Screen” was done with pre-work before class and one 90-min lab block of in-class time. The class block included a 30-min lecture and 60 min of in-class activity. The in-class activity was done in pairs.

### BIOL 459 molecular biology

Molecular Biology BIOL 459 is an upper-level course for undergraduate students at Hastings College. The course comprised of 6-students and was an elective for Biology and Biochemistry majors, with Introduction to Genetics and Cell Biology as prerequisites. It met three times a week for 80 min and twice a week for 130 min. Unlike most courses at the college, this course focuses on lab work and reading literature with a small amount of lecture material and class activities. This case was relevant to the course in understanding aspects of experimental design and data analysis. Students were introduced to the case study and worked through most of the pre-work in one 80-min period and then worked on the in-class portion the next week in an 80-min period. Students worked together in class and finished the remaining homework on their own.

### Pedagogical format

This pedagogical tool has 3 components: pre-class reading and questions (supplemental case study for students), in class lecture and activity (supplemental teaching notes and in class dataset), and post-class homework activity and questions (supplemental homework dataset and homework). The pre-class reading is a story-like description of a graduate student, Merry, joining a lab and being introduced to her first project. The narrative includes dialog between the graduate student and a senior member of the lab which explains the key topics of the case. The pre-class reading has questions embedded throughout the narrative as well as some at the end to help gage if the student is understanding the key takeaways of the reading assignment.

The in-class portion of the case study includes a lecture and in-class activity. The lecture is very customizable so lecturers could focus on the elements of the case that are most suited toward the course curriculum (supplemental teaching notes). For the implementations, lecture times typically ranged from approximately 15–30 min. Lectures were focused on what the instructor found most important for students to understand from the pre-class reading. The lecture portion included a power point that reviewed the main topics of the case (drug screening methods, cancer biology, statistical analysis methods, how quantitative polymerase chain reaction (qPCR) works, etc.) as well as time for students to ask questions about the pre-class assignment and the lecture content. The in-class activity followed the lecture and lasted anywhere between 30 min to about 2 h (over multiple class sessions). The in-class activity involved the class following the instructor through analyzing a data set using a target-based statistical metric and a metric more conducive to phenotypic screening (supplemental in class dataset). The in-class activity demonstrates what happens when you try to use a target-based metric to analyze a phenotypic screening dataset. Students used what they learned in the pre-class reading and lecture to explain which analysis made the most sense in these experiments and why. Some course instructors (PHRS 802 and 500) took time at the end of the in-class activity to discuss the biological significance of the data the class analyzed and helped students understand what the next steps would be in a drug screening campaign.

The homework assignment for the students included a second data set (supplemental homework dataset) which students were expected to analyze with both metrics to confirm the phenotypic screening metric was most appropriate. The homework assignment also included a few questions for the students to complete, to make sure they understood what their data meant in a biological context. Students were also given written step-by-step instructions on how to do the data analysis to help if they got stuck doing the homework (supplemental case study for students—last section). The homework assignment is intended to take 45–60 min to complete.

### Learning objectives

The course learning outcomes relevant to the case study state that on successful completion of the course students should be able to Table 1:

- Define phenotypic cell-based screening and identify appropriate screening controls.
- Apply statistical modeling to a phenotypic screen to identify biologically meaningful results.
- Interpret the biological significance of a Z’-value and a Z*-value.

### Data collection

Consent forms were handed out to students in the class session before the implementation to ensure they had ample time to read over the form. Study participants handed signed consent forms in to the instructor before the implementation session started.

Students were expected to have completed the pre-class reading and questions prior to the lecture to give students a foundational understanding of screening types and why they differ in the way they are statistically analyzed. Pre class questions from the reading were expected to be completed as homework before class and were turned in except for in BIOL 459 and SCI 458. Consenting participants were asked to fill out a paper survey before the lecture. The survey included questions about participant demographic and asked students to rank their level of agreement with a list of statements. The statements were focused on how comfortable the student was in describing themselves as a scientist as well as how familiar they were with the case study topics.

After the in-class activity was completed, consenting students were given a second paper survey to fill out that asked the same questions as the pre-class survey. Student pre-and post-class responses were compiled and analyzed by Wilcoxon test to determine if student understanding of the key topics improved after the lecture and in-class activity. A small multiple choice question set is provided to help further assess students pre-and post-implementation (supplemental class quizzes).

In all courses the homework dataset and questions were turned in for a grade. The homework questions were focused on assessing the students’ ability to understand the biological significance of their statistical analysis.

## Results (to date)

### Study demographics

**FIGURE 1:**
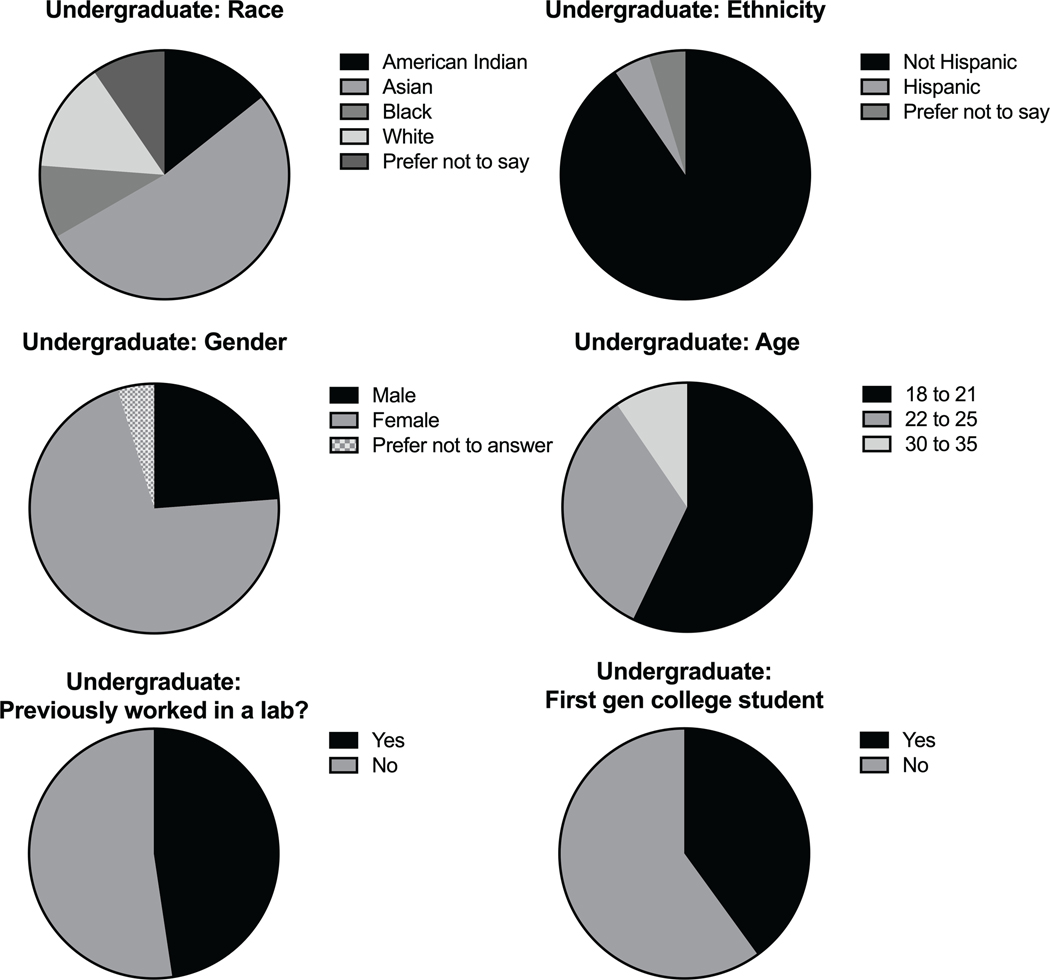
Undergraduate student demographics. Demographic breakdown of undergraduate participants in all implementations. Data was collected by survey questions given to consenting students and included multiple choice questions about the individual’s race, ethnicity, gender, and age. Students were also asked if they had previous experience in a lab setting and if they were first generation (gen) college students. Results were compiled and depicted as pie charts.

**FIGURE 2:**
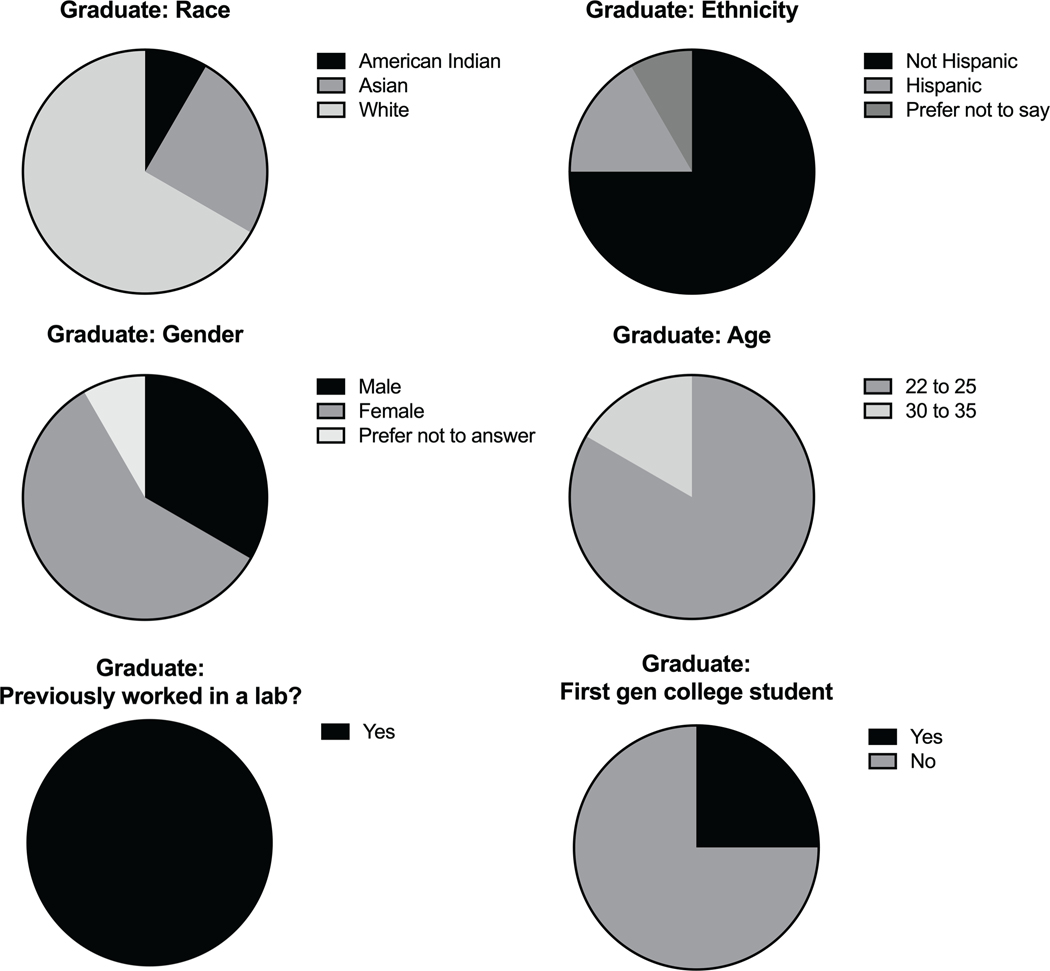
Graduate student demographics. Demographic breakdown of graduate participants from the implementation done in PHRS 802. Data was collected by survey questions given to consenting students and included multiple choice questions about the individual’s race, ethnicity, gender, and age. Students were also asked if they had previous experience in a lab setting and if they were first generation (gen) college students. Results were compiled and depicted as pie charts.

Overall, we collected data from 21 undergraduate students and 12 graduate students. The undergraduate participants were aged 18–35 with a majority (57%) of students falling in the 18–21 age range (Figure 1). Of the students surveyed, 52% had no previous lab experience and 40% were first generation college students. The majority (71%) of undergraduate participants were female, with less than 5% of students preferring not to report their gender. The graduate level participants were either in the 22–25 age range or 30–35, with most of the students (83%) aged 22–25 (Figure 2). At least 58% of the graduate students were female, with 8% preferring not to disclose their gender. Most of the graduate students (75%) were not first-generation college students. Unsurprisingly, 100% of the graduate students had previous lab experience.

### Analysis of in-class data analysis activity

**FIGURE 3:**
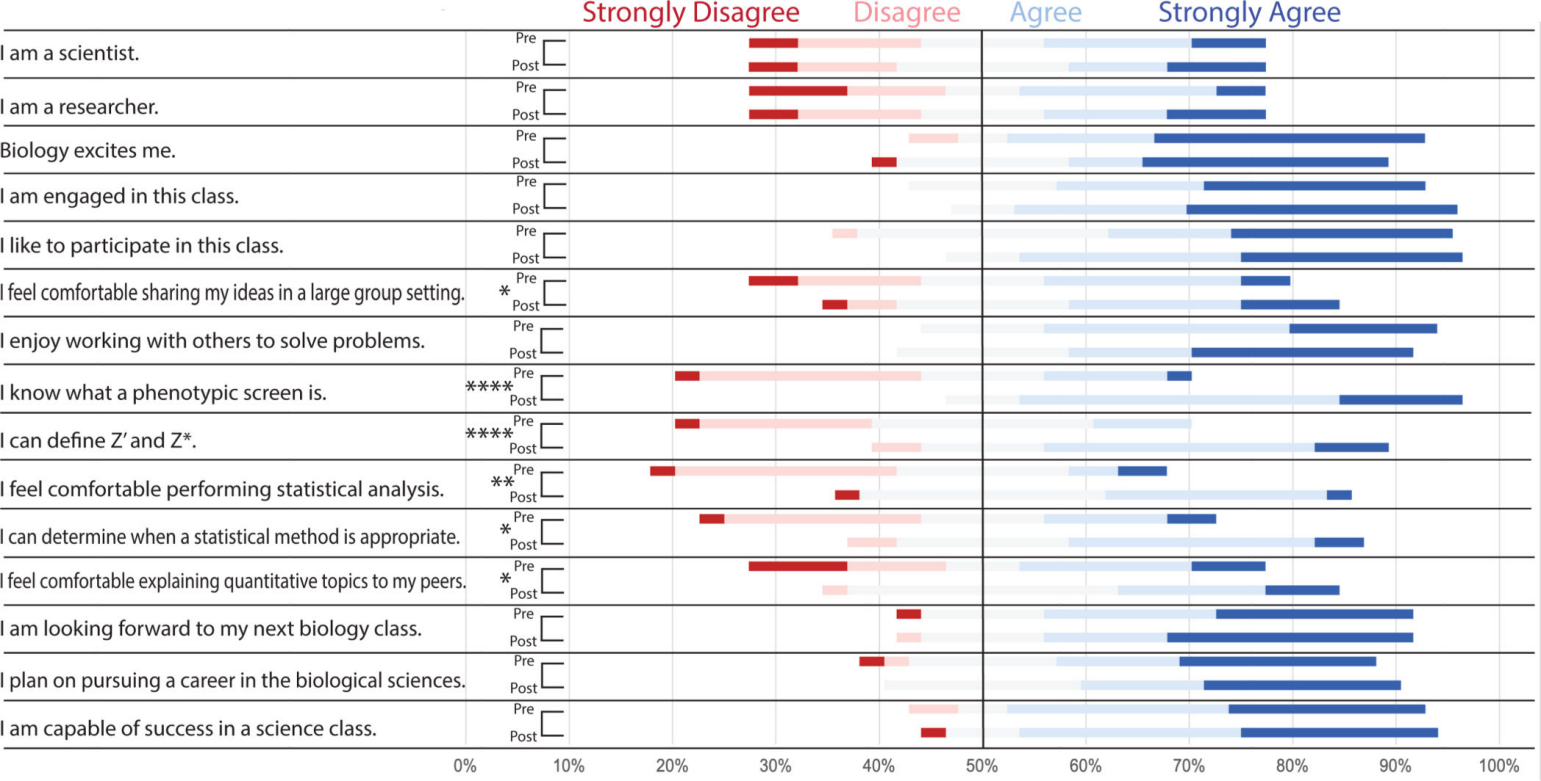
Pre-and Post-class survey questions demonstrate improvement in undergraduate student understanding of key topics. Distribution of student responses to survey questions before (pre) and after (post) the in-class portion of implementation. Students were asked to select which response (strongly agree, agree, neutral, disagree, or strongly disagree) best described their sentiment toward the statements listed. Pre-and post-class responses were analyzed via Wilcoxon test to determine if there was a significant increase in “agree” or “strongly agree” responses to any of the statements. Results suggested the case improved student comfortability sharing ideas in large groups, explaining quantitative topics, and determining appropriate statistical methods (*p < 0.05). There also was significant improvement in student comfortability in statistical analysis (**p < 0.01) as well as understanding of phenotypic screens, Z’ analysis, and Z* analysis (****p < 0.0001).

Most of the undergraduate students were interested in biology and enjoyed the course they were participating in as most students agreed or strongly agreed with the statements “biology excites me” (81%), “I am engaged in this class” (71%), and “I like to participate in this class” (67%) in the pre-class survey (Figure 3). Before the in-class lecture and statistics activity, a majority of the undergraduate students felt neutral, disagreed, or strongly disagreed with the statements “I know what a phenotypic screen is”(71.4%), “I can define Z’ and Z*”(81%), I feel comfortable performing statistical analysis”(81%), and “I can determine when a statistical method is appropriate”(66.6%). After the in-class section of the lesson plan, student responses for the statements “I know what a phenotypic screen is” and “I can define Z’ and Z*” skewed significantly more toward agree and strongly agree (*p* < 0.0001). Students also had an increased comfortability in performing statistical analysis (*p* < 0.01) as well as determining which statistical method to choose for a screening project (*p* < 0.05). We also saw a significant increase in student confidence in sharing ideas in a group setting (*p* < 0.05) as well as explaining quantitative topics to peers (*p* < 0.05).

**FIGURE 4:**
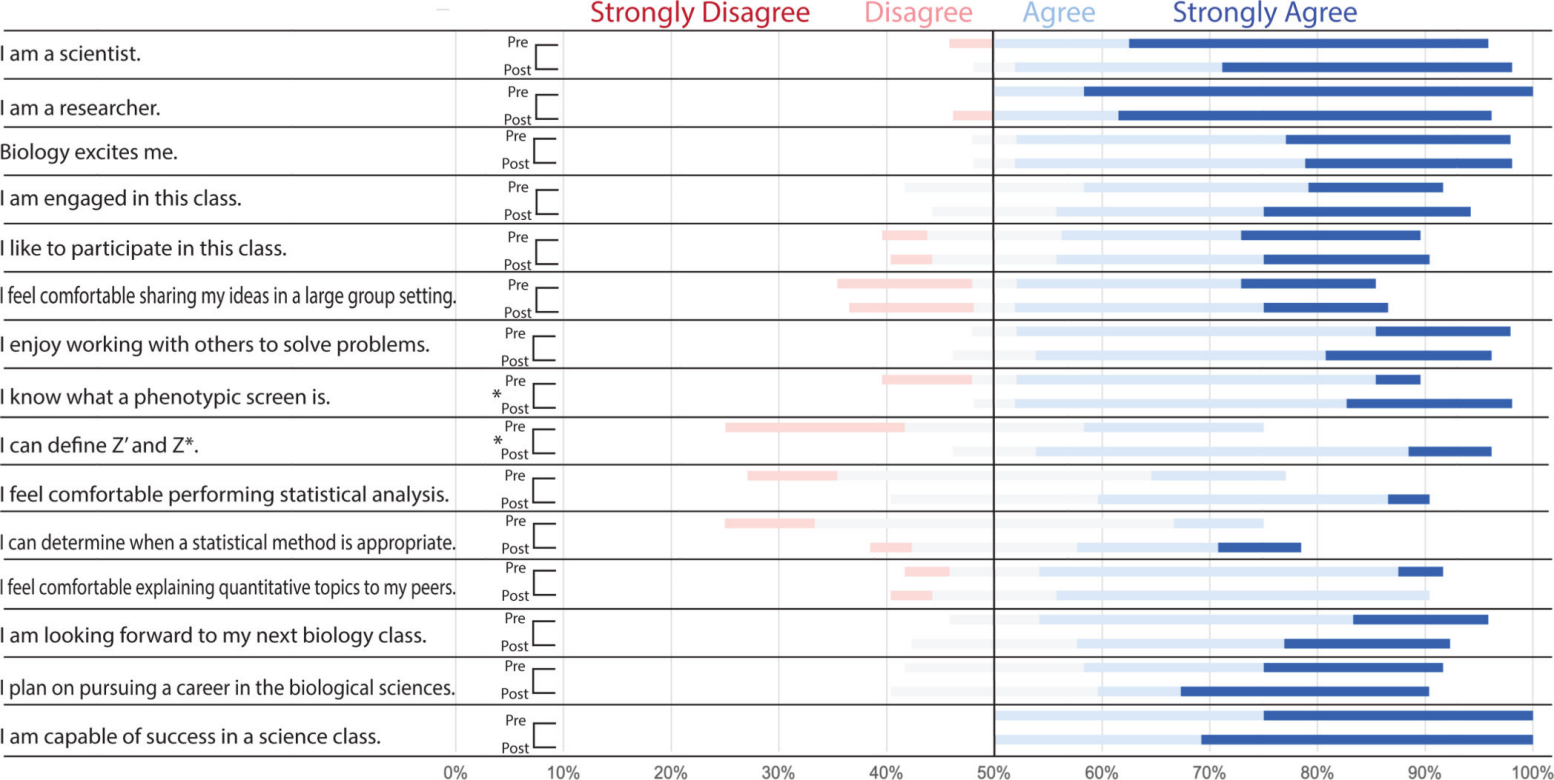
Pre-and Post-class survey questions demonstrate moderate improvement in graduate student understanding of key topics. Distribution of student responses to survey questions before (pre) and after (post) the in-class portion of implementation. Students were asked to select which response (strongly agree, agree, neutral, disagree, or strongly disagree) best described their sentiment toward the statements listed. Pre-and post-class responses were analyzed via Wilcoxon test to determine if there was a significant increase in “agree” or “strongly agree” responses to any of the statements. Results indicate the case moderately improved student understanding of phenotypic screens, Z’ analysis, and Z* analysis (*p < 0.05).

The graduate students were very comfortable with biology and most identified as scientists with most of the participants agreeing or strongly agreeing with the statements “I am a scientist” (92%), “I am a researcher” (100%), and “biology excites me” (91%) in the pre-class survey (Figure 4). The graduate students displayed a slight increase in their confidence in determining what a phenotypic screen is (*p* < 0.05) and defining Z’ and Z* (*p* < 0.05). It should be noted that the graduate student participant group was at a higher education level than the undergraduate participants. The graduate students likely had more experience in statistical analysis and quantitative topics compared to the undergraduate student population.

### Student feedback

In the post-class survey, students were asked two open-ended questions. The first question was: compared to a traditional lecture, how did the format affect your experience? The second question asked for feedback on what worked well in the in-class activity and what aspects needed to be adjusted to improve student experience. Based on student responses to the first question, it seemed that some students found the content a little hard to understand at first, but overall felt more comfortable with their quantitative skills after the activity. Many students found the lesson format more engaging and preferred the hands-on activity to traditional lectures. A selection of undergraduate and graduate student responses to question one that represent the main points are shown below:

- “I really enjoyed the flipped classroom style. I felt like I came into class with all the pieces of the puzzle but the lecturer and activity put the pieces together into a picture.”
- “It allows for more engagement with the material and gave hands-on experience in analyzing data.”
- “This was hard to understand, but it did help with computational skills. I feel more comfortable with Excel now.”
- “I enjoyed the interactive nature of examining the data. It made it more hands-on and tangible.”

Student responses to the second question mostly mentioned the length of the pre-class reading assignment and the amount of time spent on the lecture. A few students found the pre-class reading to be a little long and took a long time to read. One graduate student felt that the dialog aspect of the pre-class reading was distracting and did not contribute to their understanding. Students also suggested a shorter lecture would allow for more time to be spent on the hands-on data activity, which they felt they got more benefit from compared to the lecture. A selection of student responses that represent the main improvement suggestions have been listed below:

- “The class can be a little more interactive. Let the students do [the analysis] themselves first and then give the answer.”
- “I like this module so much, but maybe the pre-class part can be written in an easier way to [understand] because it’s a little bit difficult to understand for students [new to this topic].”
- “Maybe a bit more time build into [class] for excel because some
- [parts] move too fast.”
- “More time on hands-on example and shorter lecture.”

## Discussion

The implementations of this case demonstrate that it can be used in a wide variety of undergraduate and graduate courses to teach students topics in drug discovery research. Student survey data showed that the case was effective in improving student confidence in ability to discuss the key topics in undergraduate and graduate level courses. We had a relatively small population of participants (21 undergraduate and 12 graduate students) and we would likely get a better idea of the effectiveness of the course with a larger survey group. The undergraduate classes were small (less than 25 students), so while we were able to reach a wide range of courses, the survey data was limited. We implemented a single graduate level course, which resulted in very limited survey data for students at this level. Implementing in other graduate courses would certainly provide a better view of how the case improves graduate student understanding of drug discovery methods. We did not compare the graduate student responses to the undergraduate student responses as our sample size was too small. Understanding if there is a difference in efficacy between these two student populations would be great to assess with additional implementation data. It is also worth noting that the graduate student assessment was identical to the undergraduate student assessment, and it is possible that the case may need to be adjusted for graduate courses to improve the efficacy of the case study.

There was also some variation in how the case was implemented in each course. Most courses allotted one 50–60-min class period for

the lesson, however, one implementation was in a 90-min session and one was implemented over multiple 50 min class sessions. There was also some variety in data software used for the in-class activity. Some students preferred to use google sheets, while others used Microsoft Excel. These slight variations between implementations also factor into the limitations of this study, and more survey data may be informative for the best way to teach the case in the future.

Our implementation data shows that this in-class activity is an engaging and accessible way to teach students about drug discovery research methods. We observed throughout our implementations that many students preferred to use Google Sheets (a free resource) as they were most familiar with this software. The use of the free-ware, Google Sheets, allows students to get hands-on experience with real-world datasets at no cost to the university. The case study also includes dialog between a new graduate student and their mentors, which may be of interest to undergraduate students who are considering pursuing a graduate degree. “Behind the Screen” discusses a variety of scientific topics, making it easy for instructors to customize the case for their course. The main themes in this case are high throughput drug discovery methods, cancer biology, statistical analysis of large datasets, and ethics of data analysis and reporting.

We hope that “Behind the Screen” will be customized and implemented in multiple undergraduate courses. Each instructor that participated in this study was able to successfully tailor the case to fit into the curriculum of their course. For example, in the pharmaceutical science course implementations (PHRS 802 and PHRS 500), the lecture and in-class discussions were focused mostly on how target-based and phenotypic-based screens differ in drug discovery and when to use each type. The lecture was also dedicated to discussing the two types of statistical analyses described in the case and where and when each method would be applied. In addition to emphasizing these scientific points, the undergraduate pharmaceutical course implementation also had some discussion about graduate school, as these students were all interested in pursuing graduate careers. These classes focused less on the biology of the assay. This class also did not discuss the ethical implications that may occur when choosing statistical analysis methods.

For implementation in biology courses, it may be beneficial to focus more on the biological significance of the screen (supplemental teaching notes). This aspect will highlight the importance of understanding disease-specific cellular pathways in the design and implementation of high throughput screens. The instructor could then discuss the benefits and drawbacks of implementing phenotypic or target-based screens. Once the high throughput screening process has been introduced, methods for statistical analysis of screening data can be discussed. If ethics is part of the course curriculum, this is a great place to emphasize proper statistical procedures and discuss responsible data reporting. Following these discussions, the data from the phenotypic screen can be introduced. The instructor can explain that the assay uses qPCR to detect changes in a DNA biomarker, and “hits” (drugs that detectably change biomarker levels) are samples that fall above or below the statistical cutoff described in the case study [3 times the median of absolute deviation (MAD)] (Zhang, 2011). Lastly, we recommend making sure all students in the class understand the basics of how qPCR analysis works. In-depth methodology knowledge is not necessary, but since the in-class dataset involves working with cycle threshold (C_T_) values, it is important to ensure that students grasp the origin of these data values to gain a better understanding of statistical significance in data analysis. After the lecture, the instructor can answer any student questions and proceed to the in-class activity.

## Conclusion

High throughput screening commonly used in drug discovery campaigns, and while it is essential that students are taught how to analyze large datasets, it is difficult for undergraduate institutions to provide hands-on experience with these methods. “Behind the Screen” is an interactive and highly versatile case study that provides students the opportunity to work with large datasets modeled from a real-world first-in-class screen. The case aims to increase students’ confidence in their ability to define phenotypic screening and proper controls, apply statical modeling to a phenotypic screen, and interpret the biological significance of a Z’- and Z*- value. Implementations of this case proved it to be successful in significantly improving undergraduate and graduate confidence in ability to confidently discuss the learning objectives. While the case was effective in these student populations, our sample sizes were small. Further implementation will allow us to evaluate if the case performs differently between undergraduate and graduate students.

## Supplementary Material

Presentation 1

Table 1

Table 2

Table 3

Table 4

Table 5

Table 6

Table 7

Table 8

Table 9

Table 10

## Figures and Tables

**TABLE 1:** Breakdown of where each learning objective is addressed in the case materials.

Learning objective	Case study materials addressing objective
Define phenotypic cell-based screening and identify appropriate screening controls	• Case study for students pre-work parts 1 and 2 • Pre- and post-class quiz questions
Apply statistical modeling to a phenotypic screen to identify biologically meaningful results	• Case study for students pre-work parts 2 and 3 • In-class and homework data analysis activities • Homework questions
Interpret the biological significance of a Z-value and a Z*-value	• Case study for students part 3 • In-class and homework data analysis activities • Homework questions

All case materials can be found in the supplemental documents provided.

## Data Availability

The raw data supporting the conclusions of this article will be made available by the authors, without undue reservation.
