# Supplementary material for: Behind the screen: drug discovery using the big data of phenotypic analysis
Source: Front Educ (Lausanne). Author manuscript; Available in PMC 2024 Sep 5. (PMC11376653; doi:10.3389/feduc.2024.1342378)
Supplement: Presentation 1 [file NIHMS1969654-supplement-Presentation_1.pptx]

## Slide 1
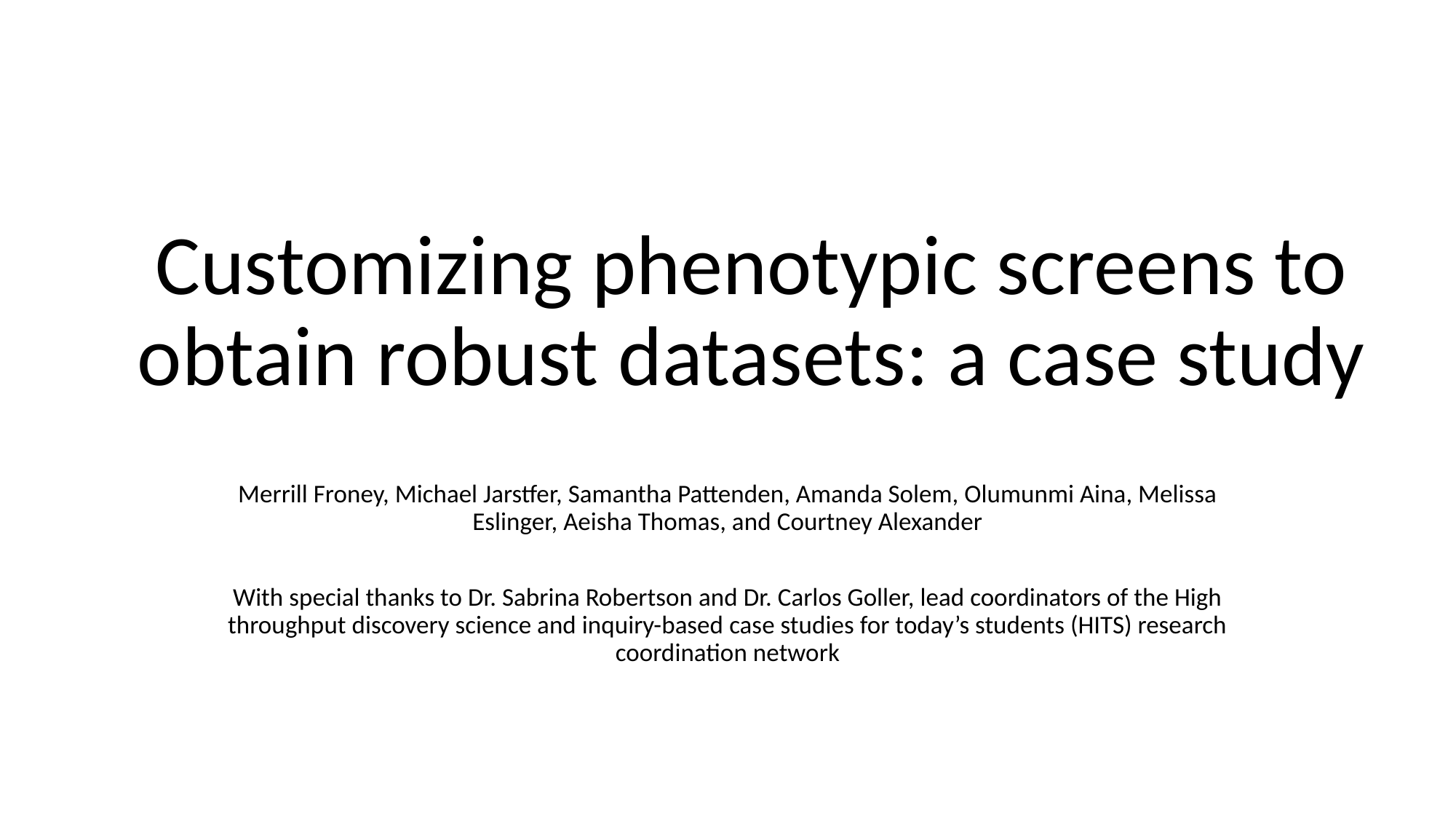

# Customizing phenotypic screens to obtain robust datasets: a case study
Merrill Froney, Michael Jarstfer, Samantha Pattenden, Amanda Solem, Olumunmi Aina, Melissa Eslinger, Aeisha Thomas, and Courtney Alexander
With special thanks to Dr. Sabrina Robertson and Dr. Carlos Goller, lead coordinators of the High throughput discovery science and inquiry-based case studies for today’s students (HITS) research coordination network

## Slide 2
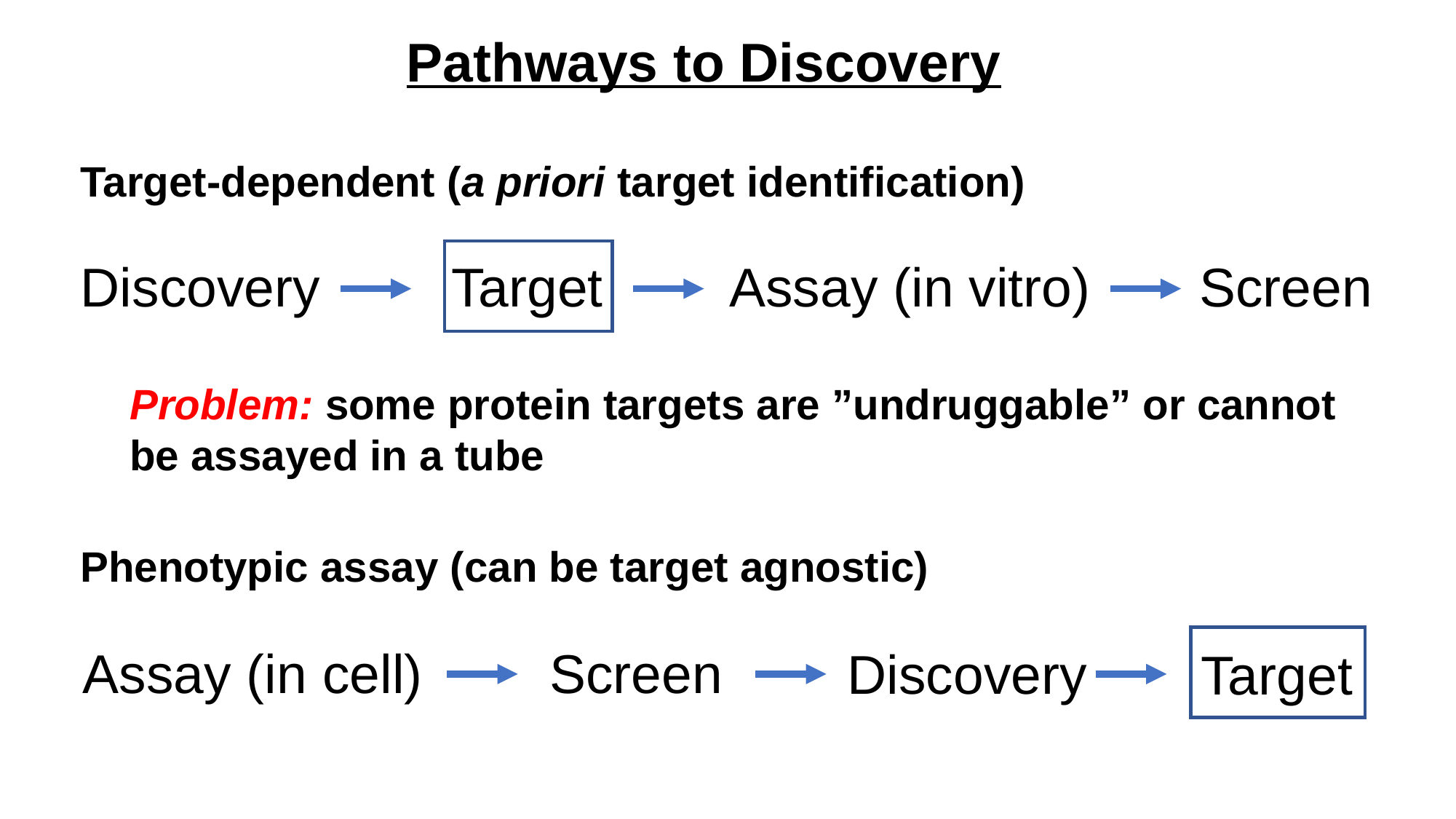

# Pathways to Discovery
Target-dependent (a priori target identification)
Discovery
Target
Assay (in vitro)
Screen
Problem: some protein targets are ”undruggable” or cannot be assayed in a tube
Phenotypic assay (can be target agnostic)
Screen
Discovery
Target
Assay (in cell)

## Slide 3
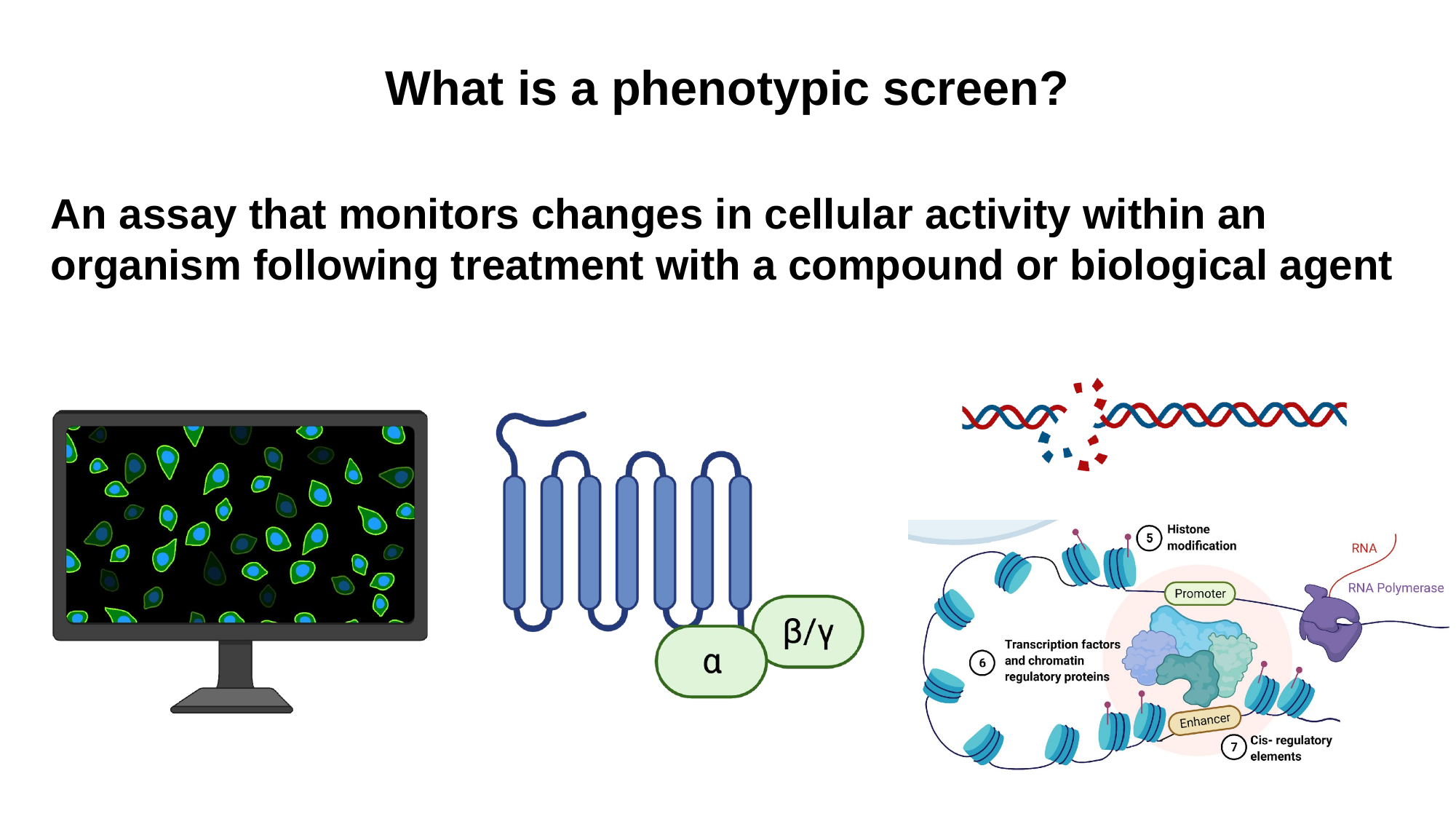

What is a phenotypic screen?
An assay that monitors changes in cellular activity within an organism following treatment with a compound or biological agent

## Slide 4
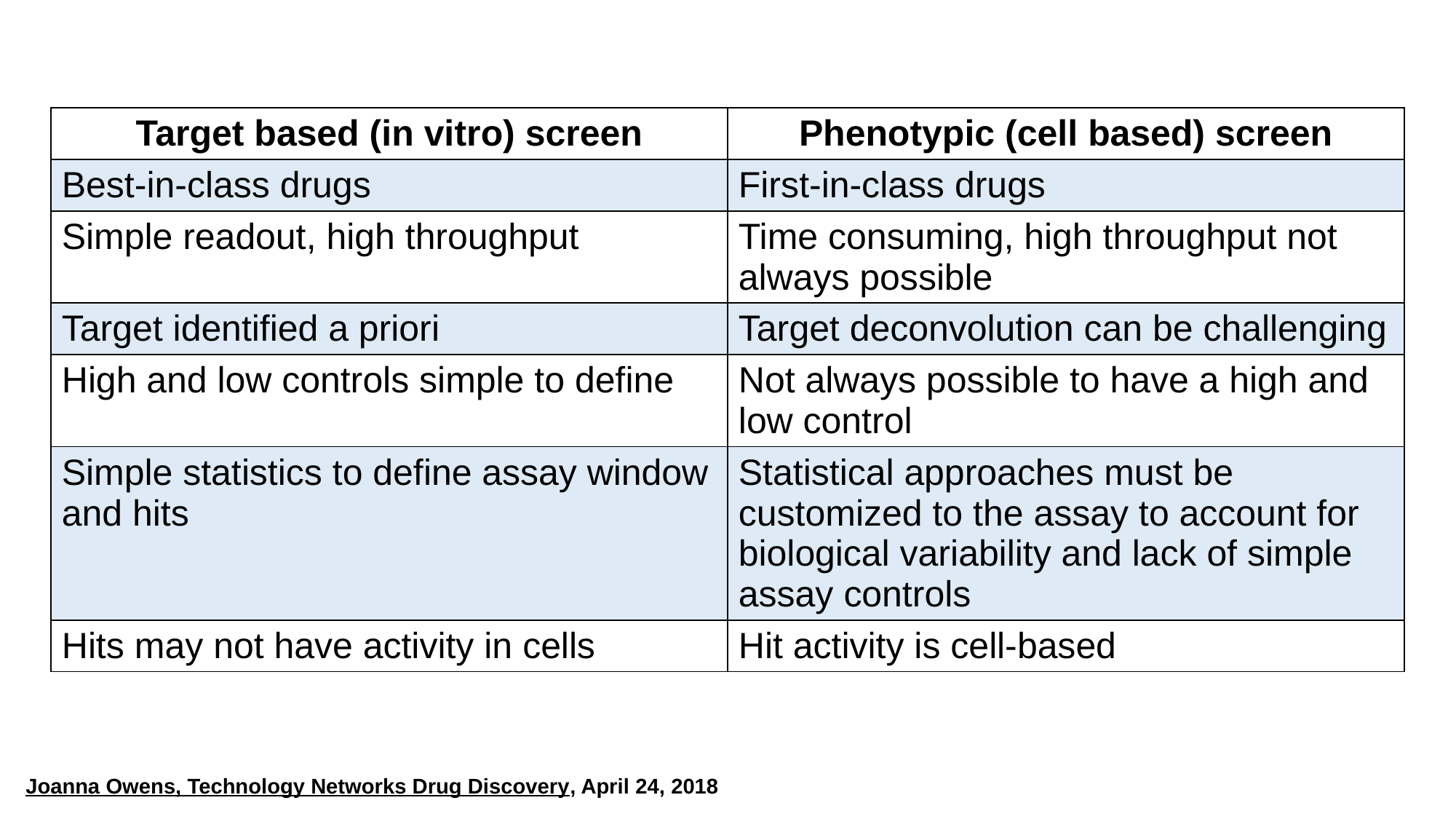

| Target based (in vitro) screen | Phenotypic (cell based) screen |
| --- | --- |
| Best-in-class drugs | First-in-class drugs |
| Simple readout, high throughput | Time consuming, high throughput not always possible |
| Target identified a priori | Target deconvolution can be challenging |
| High and low controls simple to define | Not always possible to have a high and low control |
| Simple statistics to define assay window and hits | Statistical approaches must be customized to the assay to account for biological variability and lack of simple assay controls |
| Hits may not have activity in cells | Hit activity is cell-based |
Joanna Owens, Technology Networks Drug Discovery, April 24, 2018

## Slide 5
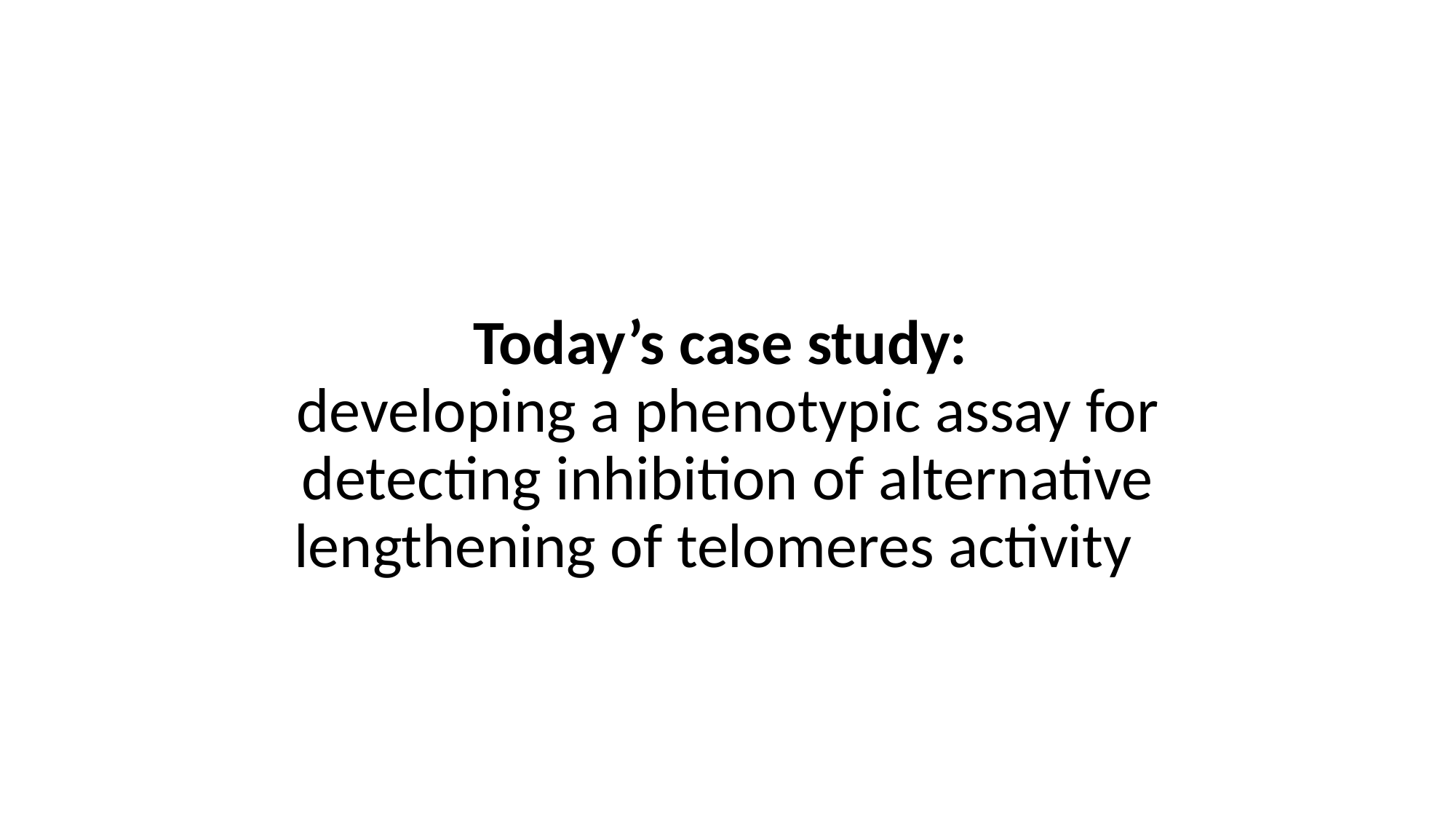

# Today’s case study: developing a phenotypic assay for detecting inhibition of alternative lengthening of telomeres activity

## Slide 6
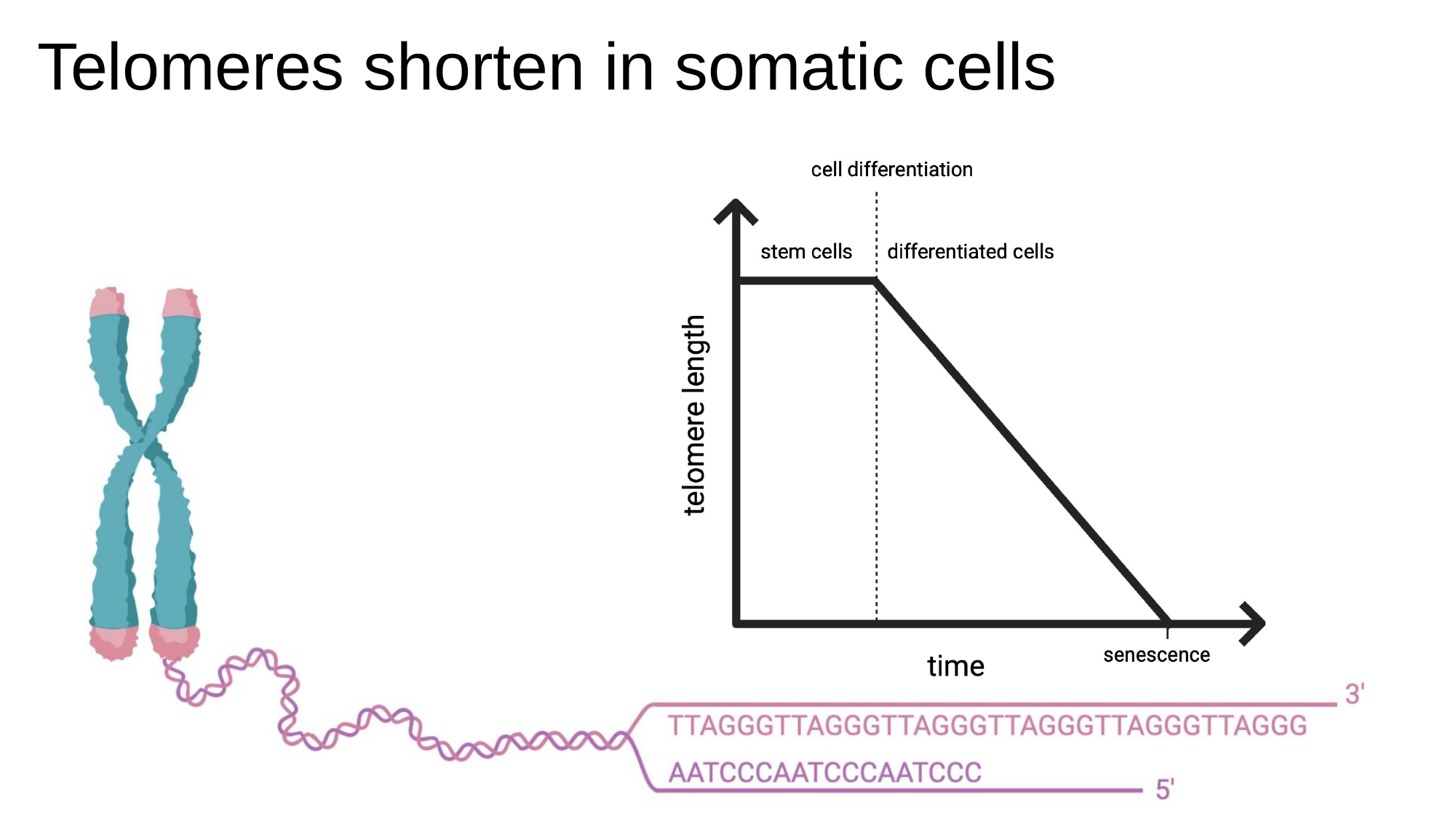

Telomeres shorten in somatic cells

## Slide 7
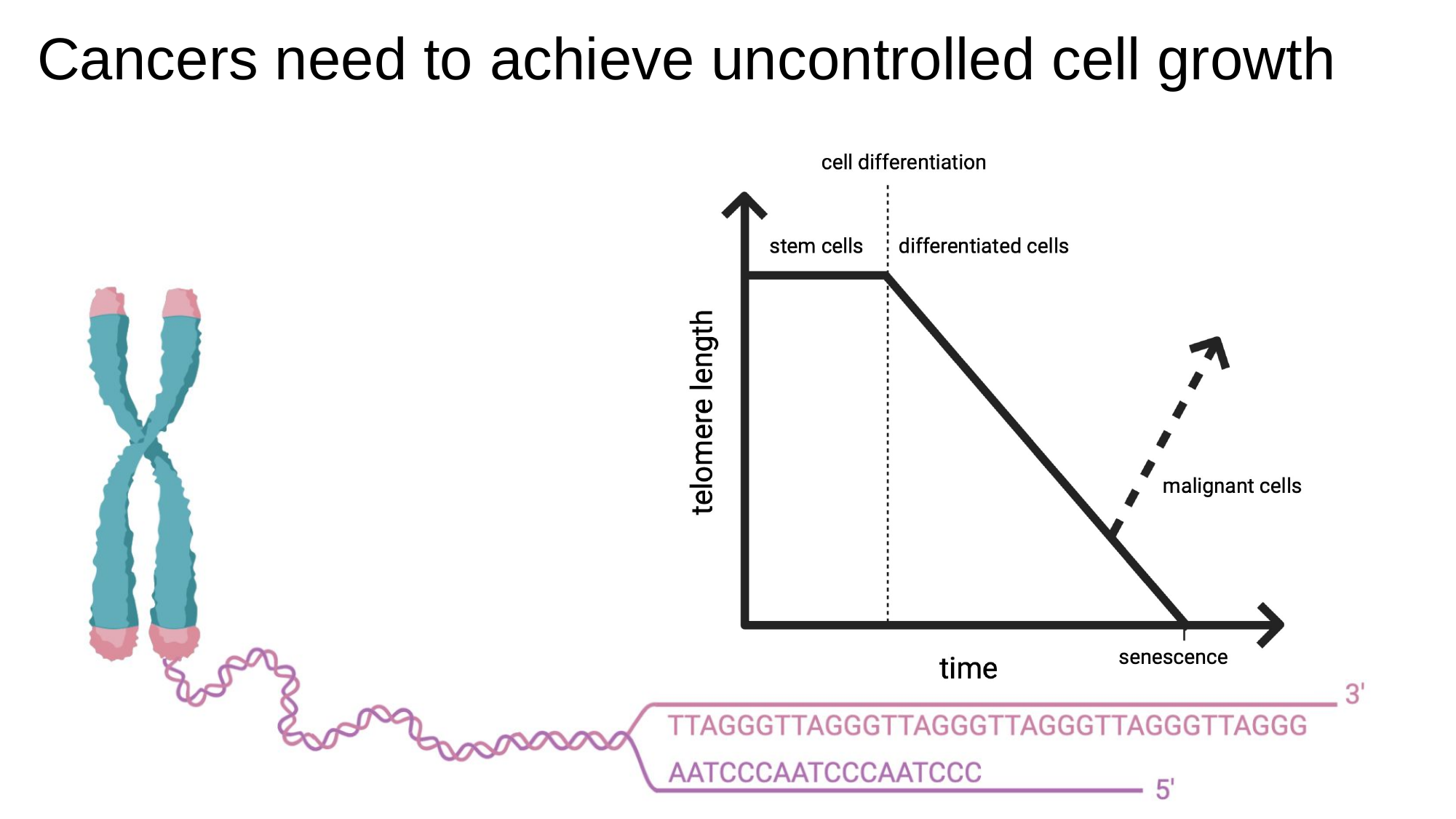

Cancers need to achieve uncontrolled cell growth

## Slide 8
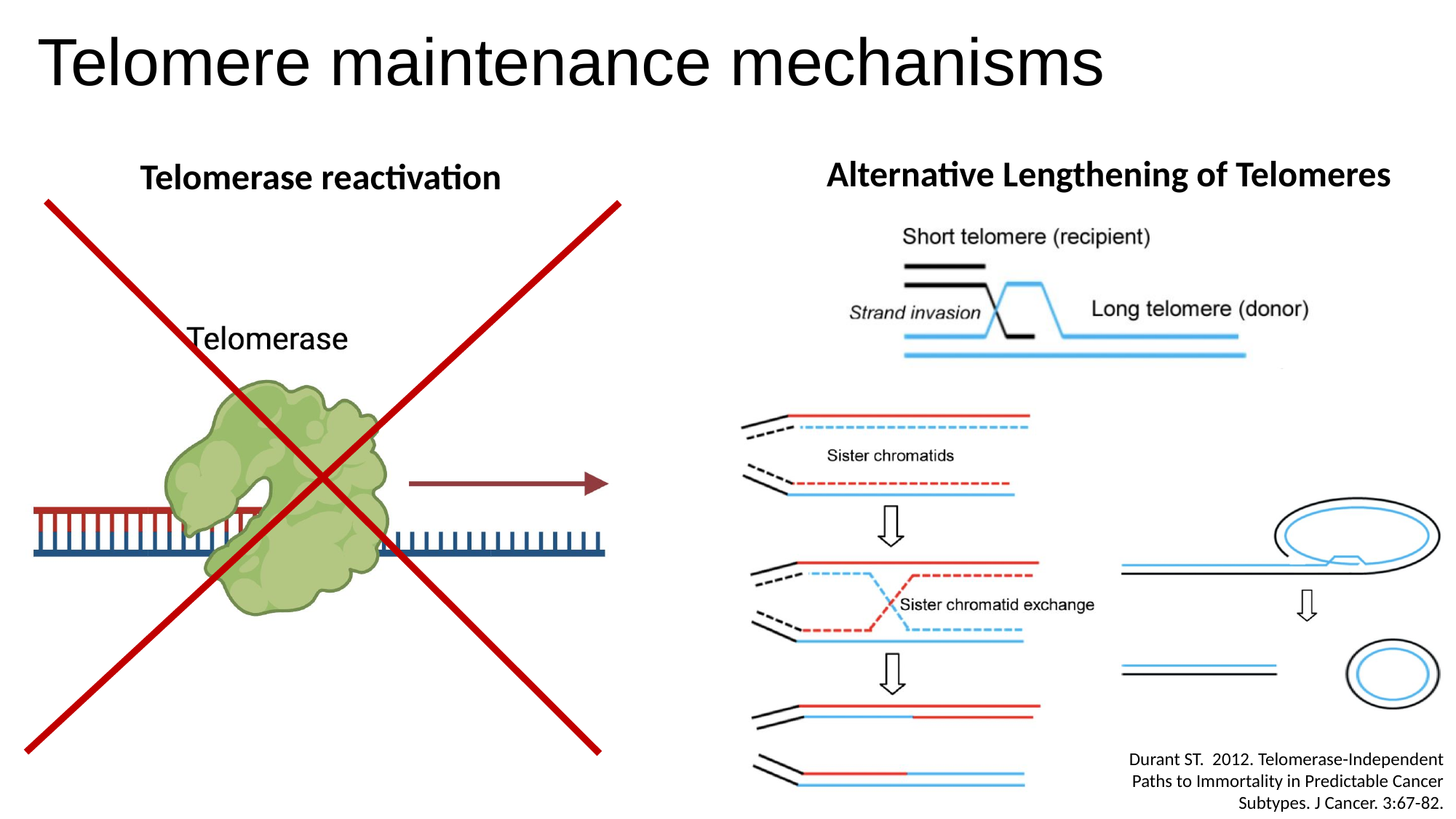

Telomere maintenance mechanisms
Alternative Lengthening of Telomeres
Telomerase reactivation
Durant ST. 2012. Telomerase-Independent Paths to Immortality in Predictable Cancer Subtypes. J Cancer. 3:67-82.

## Slide 9
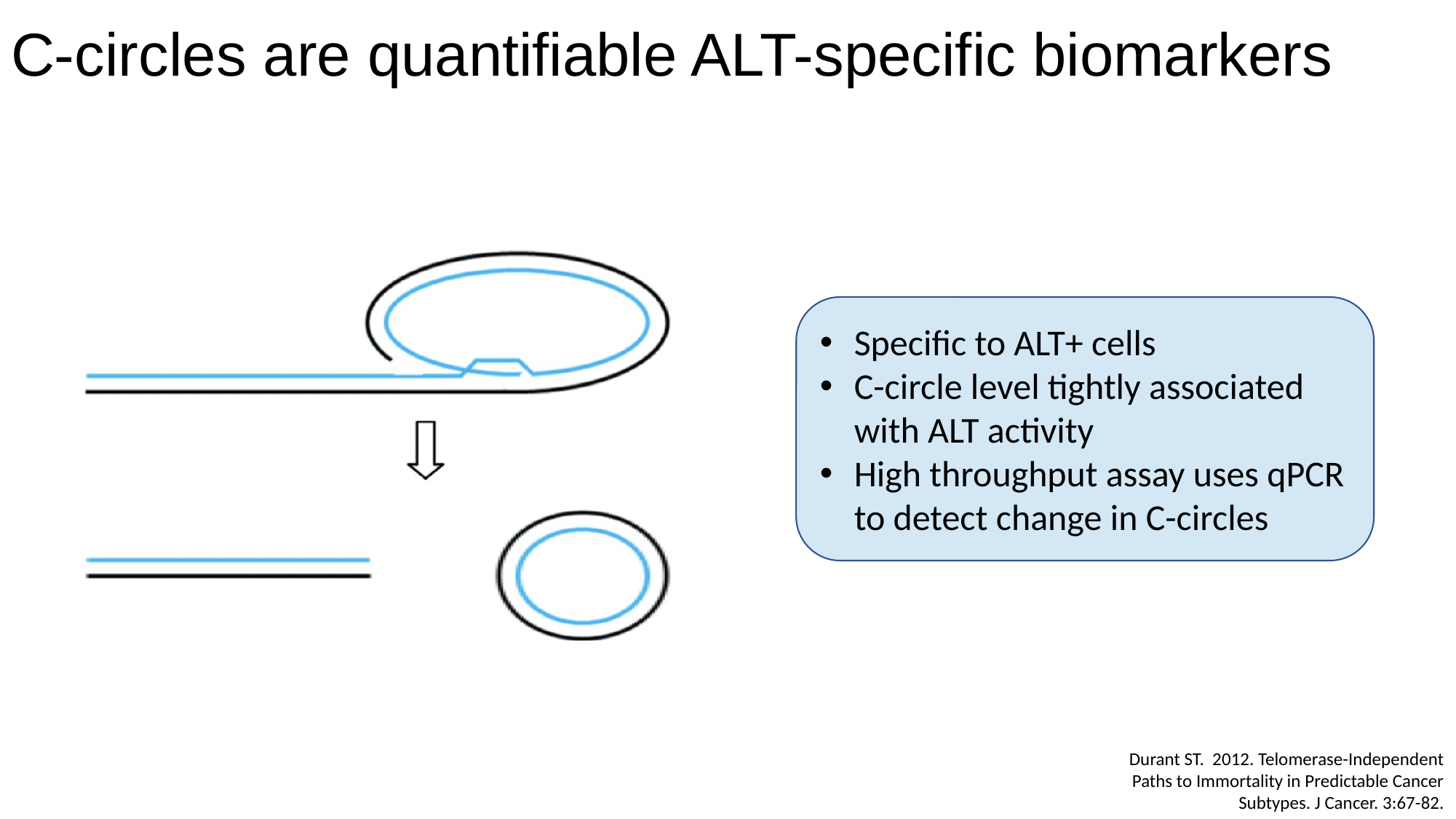

C-circles are quantifiable ALT-specific biomarkers
Specific to ALT+ cells
C-circle level tightly associated with ALT activity
High throughput assay uses qPCR to detect change in C-circles
Durant ST. 2012. Telomerase-Independent Paths to Immortality in Predictable Cancer Subtypes. J Cancer. 3:67-82.

## Slide 10
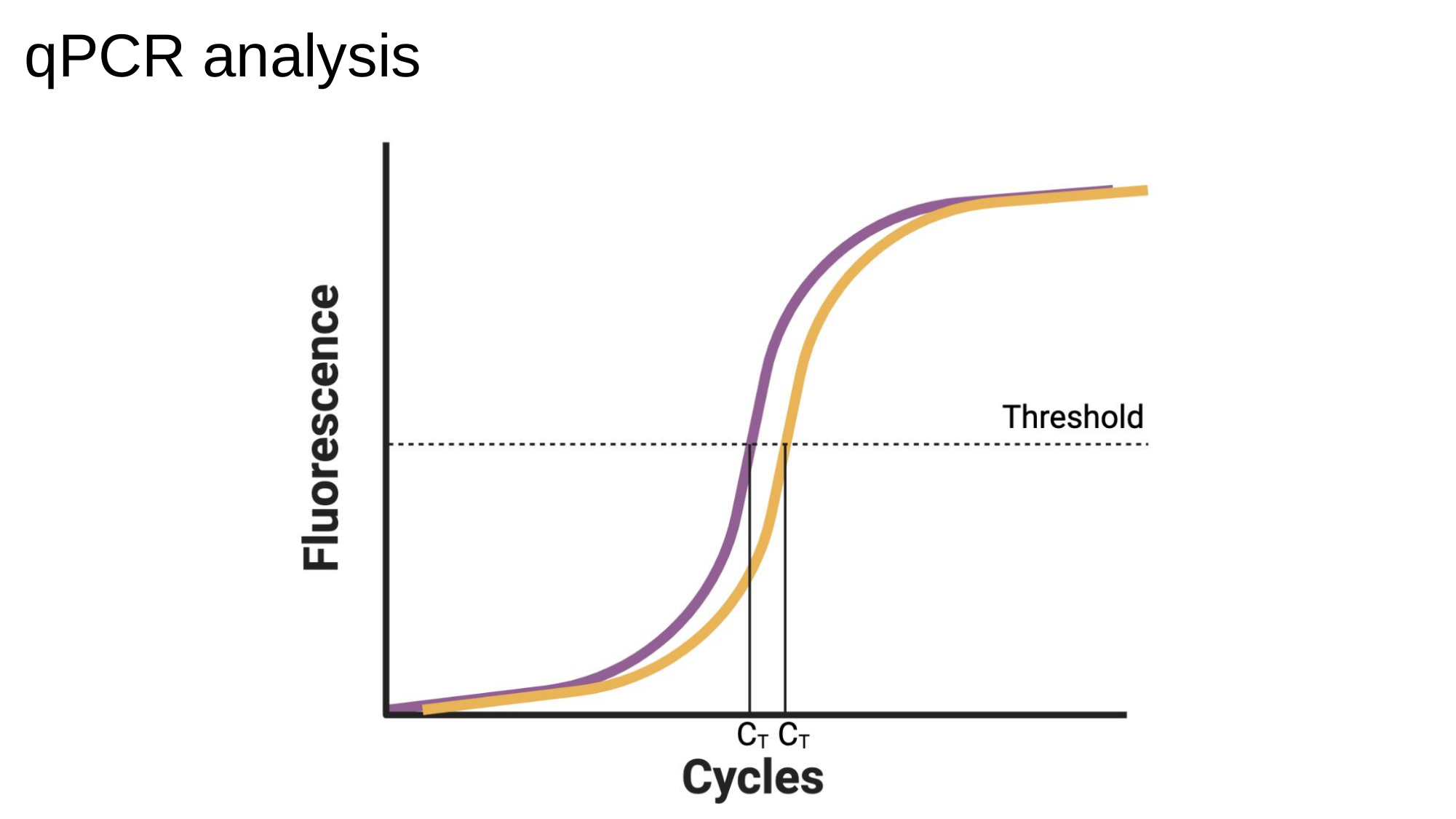

qPCR analysis

## Slide 11
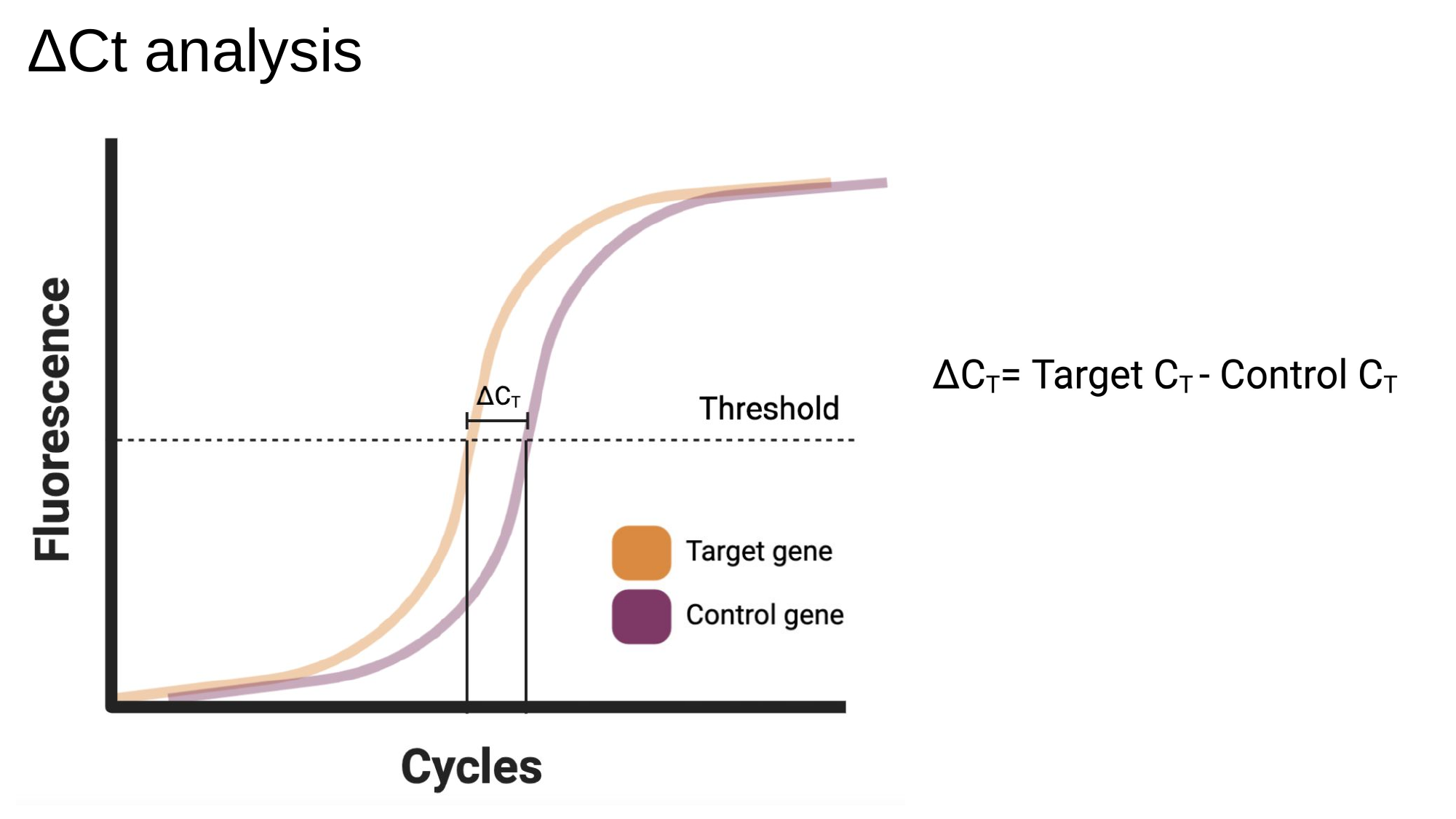

ΔCt analysis

## Slide 12
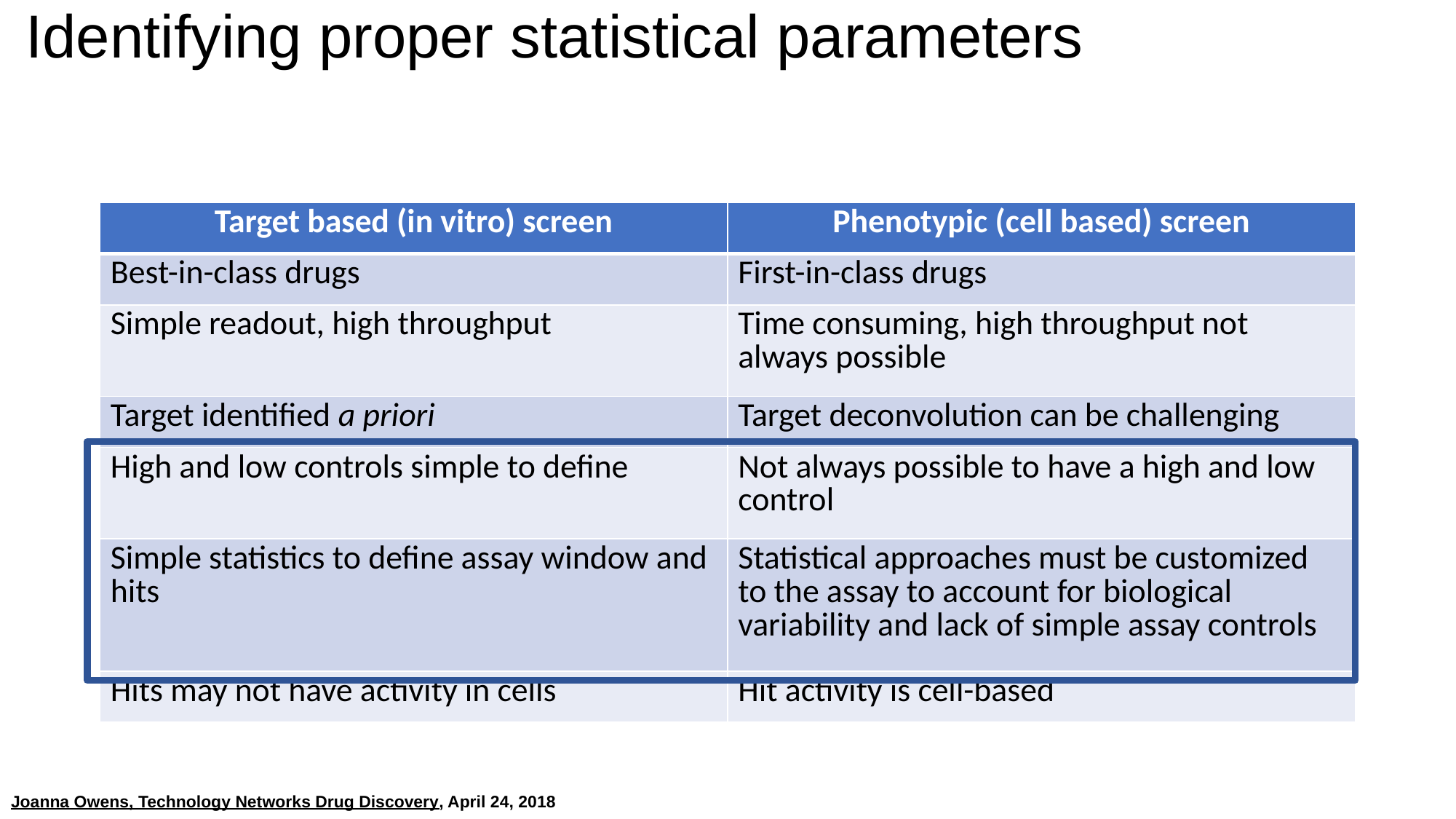

Identifying proper statistical parameters
| Target based (in vitro) screen | Phenotypic (cell based) screen |
| --- | --- |
| Best-in-class drugs | First-in-class drugs |
| Simple readout, high throughput | Time consuming, high throughput not always possible |
| Target identified a priori | Target deconvolution can be challenging |
| High and low controls simple to define | Not always possible to have a high and low control |
| Simple statistics to define assay window and hits | Statistical approaches must be customized to the assay to account for biological variability and lack of simple assay controls |
| Hits may not have activity in cells | Hit activity is cell-based |
Joanna Owens, Technology Networks Drug Discovery, April 24, 2018

## Slide 13
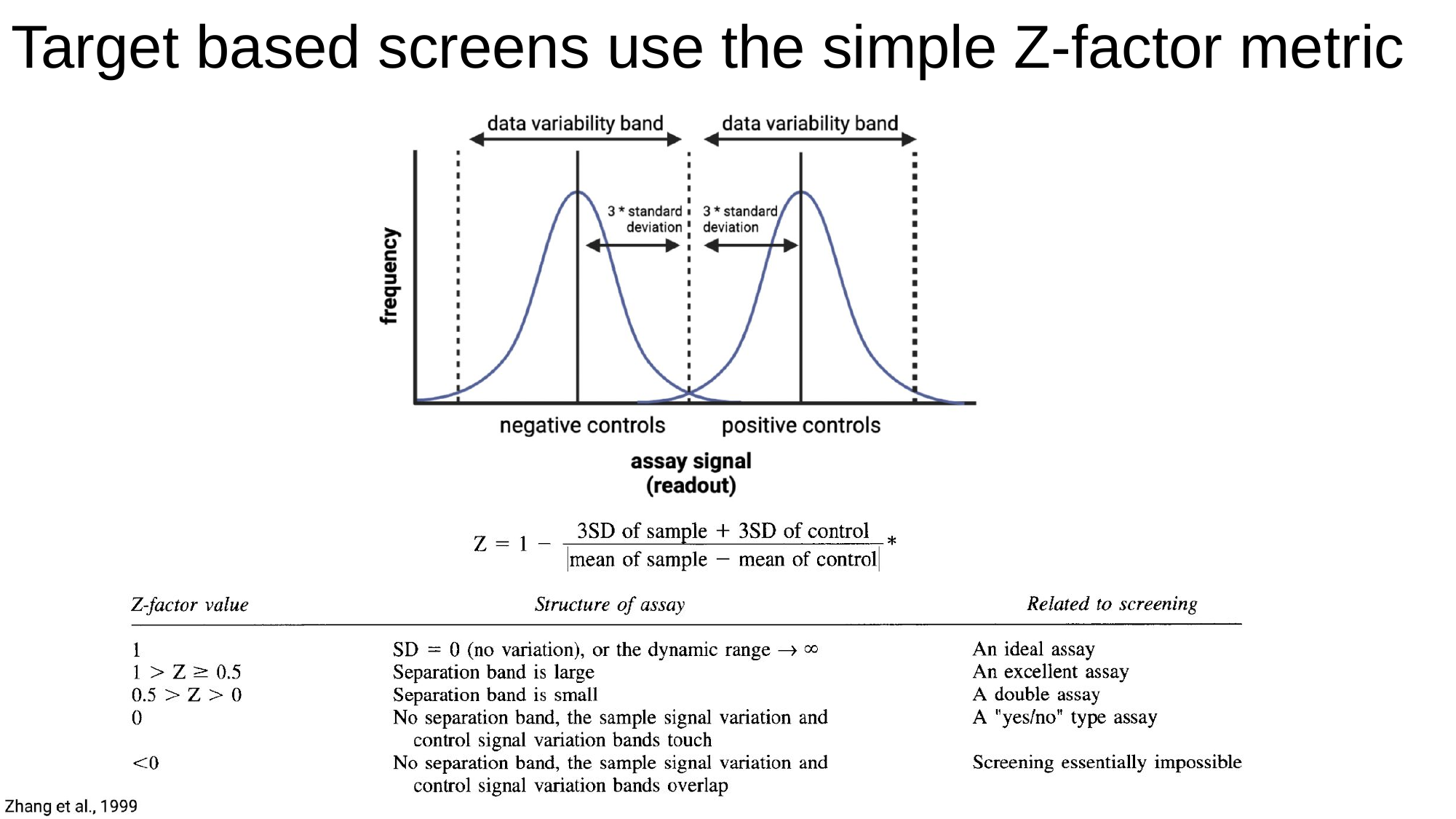

Target based screens use the simple Z-factor metric

## Slide 14
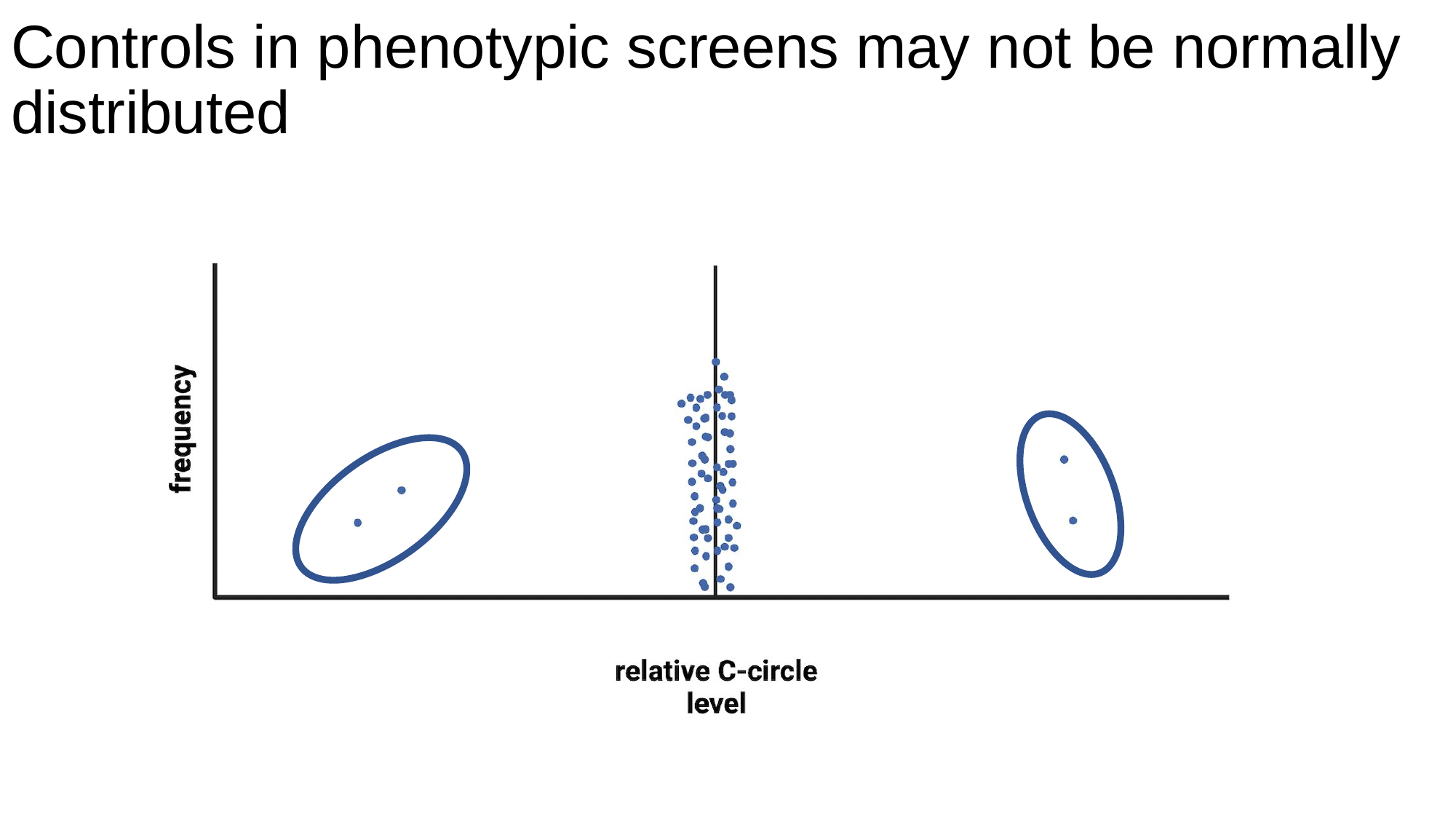

Controls in phenotypic screens may not be normally distributed

## Slide 15
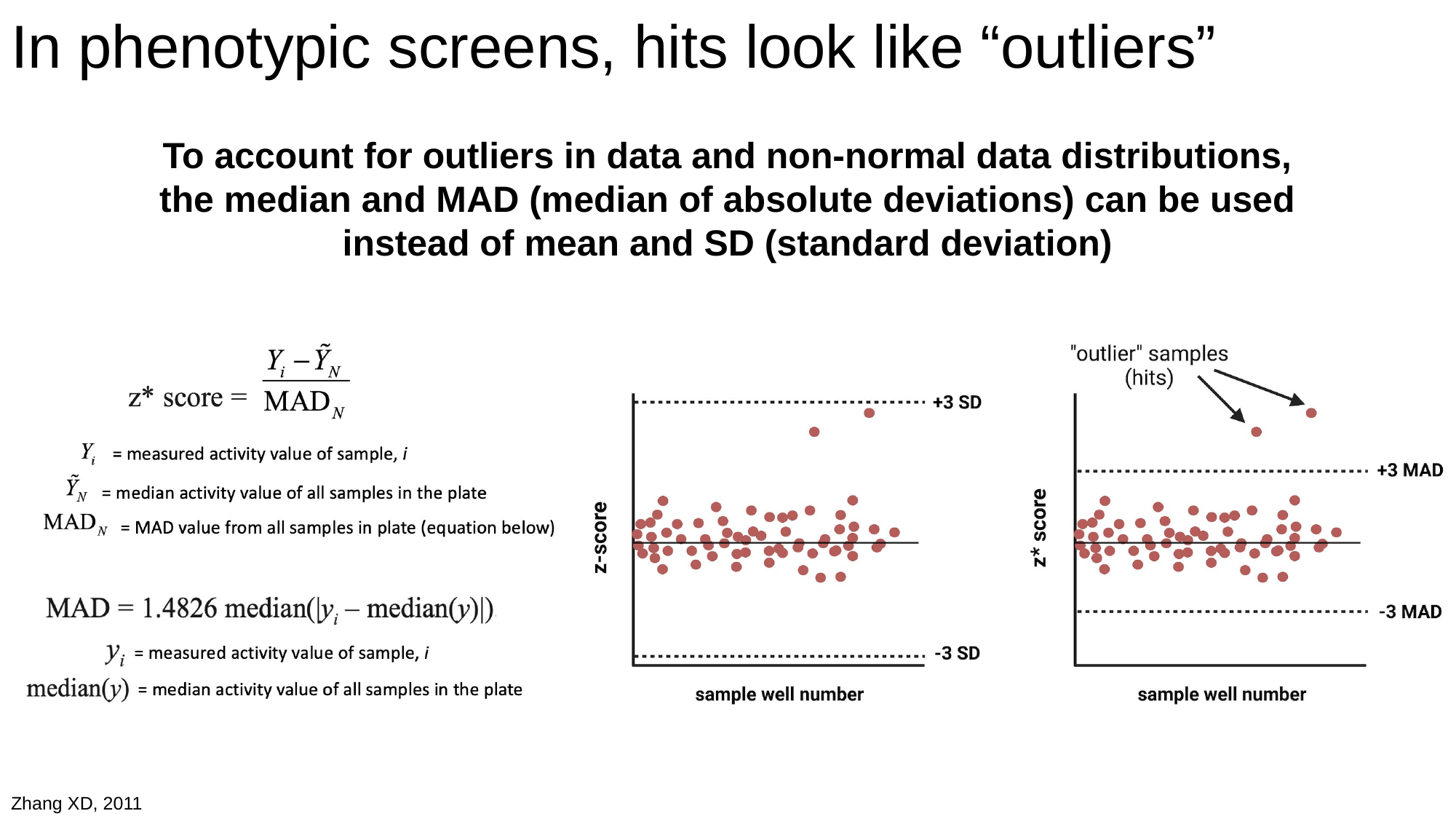

In phenotypic screens, hits look like “outliers”
To account for outliers in data and non-normal data distributions, the median and MAD (median of absolute deviations) can be used instead of mean and SD (standard deviation)
Zhang XD, 2011

## Slide 16
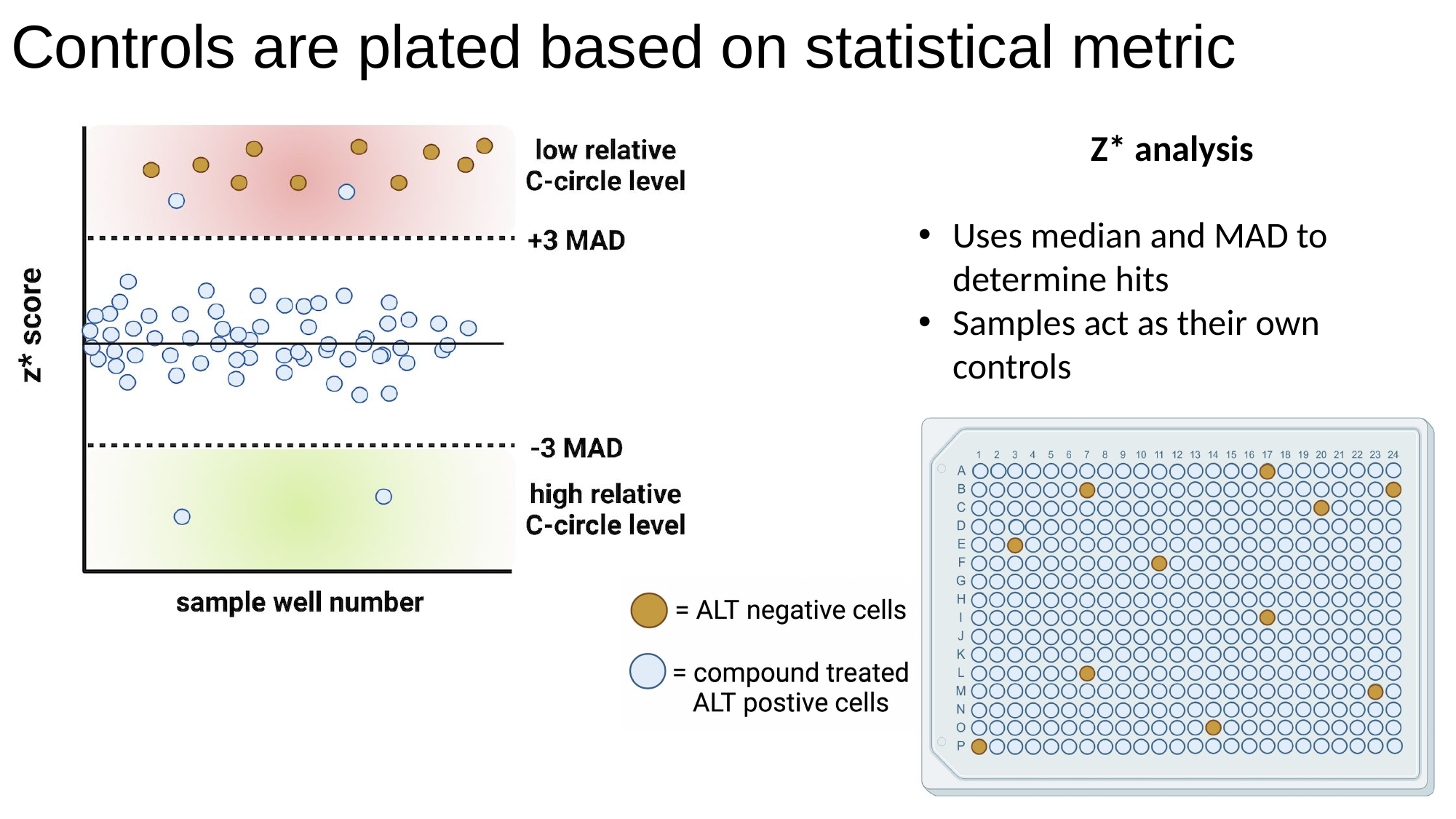

Controls are plated based on statistical metric
Z* analysis
Uses median and MAD to determine hits
Samples act as their own controls

## Slide 17
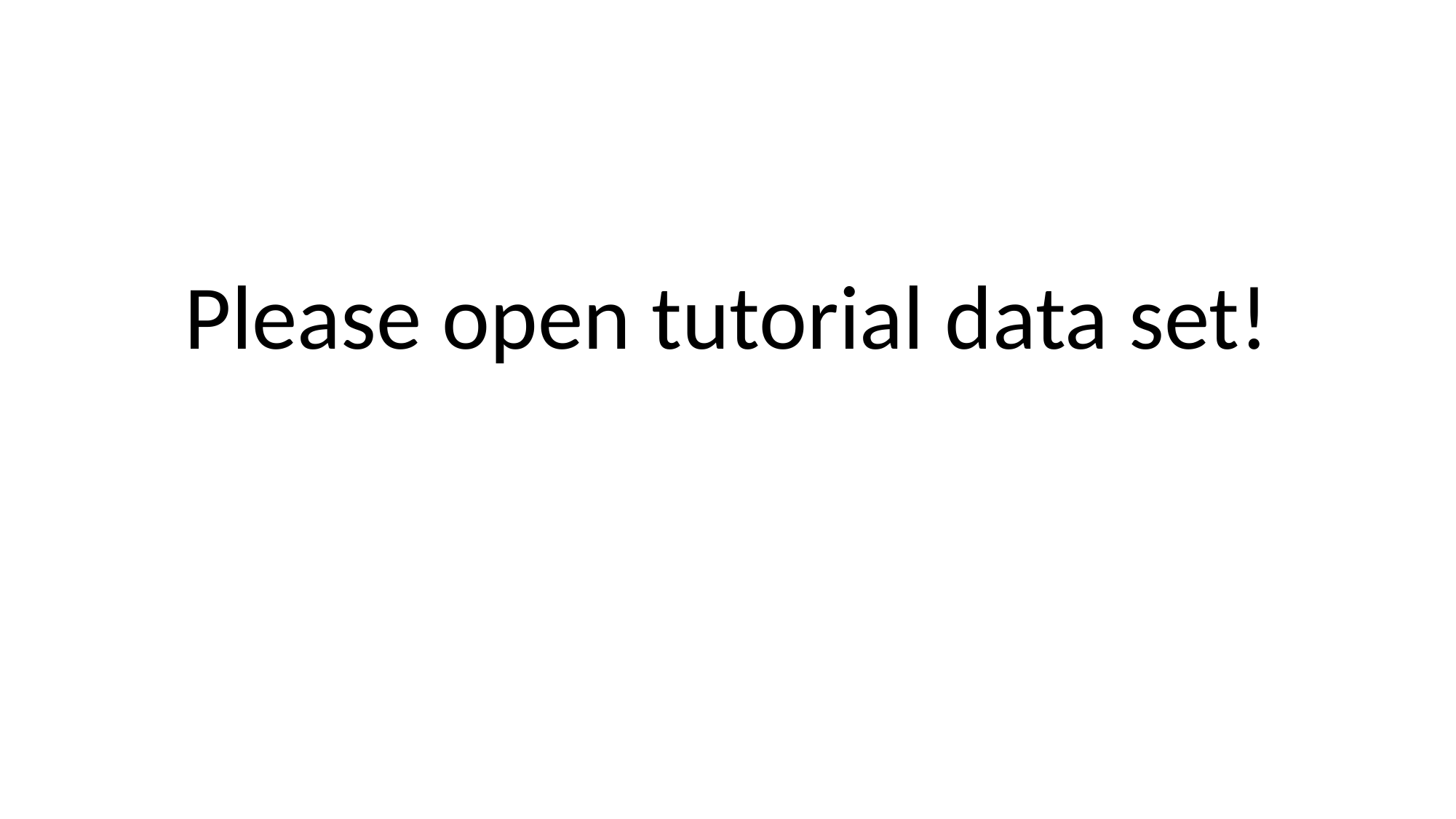

# Please open tutorial data set!
