# Supplementary material for: Behind the screen: drug discovery using the big data of phenotypic analysis
Source: Front Educ (Lausanne). Author manuscript; Available in PMC 2024 Sep 5. (PMC11376653; doi:10.3389/feduc.2024.1342378)
Supplement: Table 3 [file NIHMS1969654-supplement-Table_3.docx]

**GIVE TO STUDENTS AT BEGINNING AND END OF CLASS**

**Quiz**

1. If you’re setting up a screen to find a better drug that inhibits the protein Plnk1, you should probably do:
   - 1. **A target-based screen with Z’ analysis and positive and negative controls.**
     2. A phenotypic screen with  Z’ analysis and positive and negative controls.
     3. A target-based screen with z* analysis and negative controls.
     4. A phenotypic screen with z* analysis and negative controls.

1. If you want to set up a screen to find a drug to treat the rare disease Wilts whose cause is unclear, you should probably do:
2. A target-based screen with Z’ analysis and positive and negative controls.
3. A phenotypic screen with  Z’ analysis and positive and negative controls.
4. A target-based screen with z* analysis and negative controls.
5. **A phenotypic screen with z* analysis and negative controls.**

1. Phenotypic and target-based screens require different statistical methods to evaluate hits because
2. You should always try every method until you get the number of hits your teacher asked for.
3. One is for the best-in-class drug while the other is first-in-class so they cannot be evaluated the same way.
4. **They typically produce data sets with a particular distribution - your statistical analysis must match the distribution of points (bell curve vs non-bell curve).**
5. The detection method is different for target-based vs phenotypic screens - the statistical analysis depends on what type of instrument collected the data.
