# Supplementary material for: Behind the screen: drug discovery using the big data of phenotypic analysis
Source: Front Educ (Lausanne). Author manuscript; Available in PMC 2024 Sep 5. (PMC11376653; doi:10.3389/feduc.2024.1342378)
Supplement: Table 5 [file NIHMS1969654-supplement-Table_5.docx]

**HOMEWORK**

Merry is anxious to analyze the next 300 compounds in the library.  She downloads the homework dataset file to prepare for analysis.  Using these results, what are the (Z’, Z*, target drugs)

**Z Score Calculations**

|  | Average | SD | 3XSD |
| --- | --- | --- | --- |
| Untreated |  |  |  |
| Negative Control |  |  |  |

Z score  _____________  In one sentence, what does this mean about the phenotypic screen results?

**Z* Calculations**

| Median |  |
| --- | --- |
| MAD |  |

Z* score ___________________   In one sentence, what does this mean about the phenotypic screen results?

Where are the negative control samples on the your graph? Is this where you expect a sample with no C-circles to be? Explain in 1-2 sentences why or why not.

Using the analyzed data set, what recommendations does Merry make to her PI regarding potential drugs of interest?  Has Merry identified any drug hits or should more be screened?  In your response, make sure that you include what statistic(s) were used and your rationale.
