# Supplementary material for: Behind the screen: drug discovery using the big data of phenotypic analysis
Source: Front Educ (Lausanne). Author manuscript; Available in PMC 2024 Sep 5. (PMC11376653; doi:10.3389/feduc.2024.1342378)
Supplement: Table 8 [file NIHMS1969654-supplement-Table_8.docx]

**RUBRIC FOR GRADING HOMEWORK**

|  | 3 | 2 | 1 |
| --- | --- | --- | --- |
| Calculations Correct | No error | 1-2 errors | Multiple errors |
| Correct statistic used | Yes |  | No |
| Statistic used properly | Appropriately |  | Inappropriately |
| Recommendation rationale | Explained well |  |  |

**Z’ and Z* answers can be found in the homework dataset answer key**

Q: In one sentence, what does the Z’ score mean about the phenotypic screen results?

The Z’ score implies that the screening window is not large enough to accurately detect hits using a Z’ analysis. This is likely due to the fact that the phenotypic screen does not have normally distributed data and a set of controls compatible with this type of analysis.

Q: In one sentence, what does the Z* data mean about the phenotypic screen results?

The Z* data shows that this is a more accurate way to analyze this dataset because of the parameters of the screening experiment.

Q: Where are the negative control samples on the your graph? Is this where you expect a sample with no C-circles to be? Explain in 1-2 sentences why or why not.

Negative controls fall above 3MAD. This is where they should fall since in a Z* analysis the controls with lower C-circle level fall above 3MAD.

Q: Using the analyzed data set, what recommendations does Merry make to her PI regarding potential drugs of interest?  Has Merry identified any drug hits or should more be screened?  In your response, make sure that you include what statistic(s) were used and your rationale.

Merry should suggest further investigation of drugs that fell above 3MAD as this implies these drugs may be inhibiting ALT (because they resulted in a decrease in C-circles). Yes she has.

**ADDITIONAL SUMMARY QUESTIONS ON HOW APPLIES OR RELATES TO ALT**

Q: Propose the next steps of research.

If Merry were successful and identifies a drug, what are her next steps?

Next steps would be to validate or confirm her hit compounds. She would want to run these in her assay again in replicates to make sure the change is consistently seen across multiple biological replicates. Once she has confirmed the drugs have activity against ALT cells, she should plan experiments that determine how the protein target of the drug is important to ALT activity.

Q: In your own words, explain how this phenotypic screen is helpful in the fight against cancer. (1-2 sentences)

Any response about how the screen helps identify new key proteins in the ALT pathway that can help drive development of ALT-specific drugs.
