# Supplementary material for: Behind the screen: drug discovery using the big data of phenotypic analysis
Source: Front Educ (Lausanne). Author manuscript; Available in PMC 2024 Sep 5. (PMC11376653; doi:10.3389/feduc.2024.1342378)
Supplement: Table 9 [file NIHMS1969654-supplement-Table_9.docx]

**Teaching Notes**

**Behind the Screen: Drug Discovery using the Big Data of Phenotypic Analysis**

**INTRODUCTION/BACKGROUND**

This case is a learning tool that allows instructors to teach students about current methods in pharmaceutical research in a hands-on and accessible manner. By the end of this lesson plan, students should be able to describe the difference between target-based and phenotypic-based high throughput screening methods, determine the biological significance of a phenotypic-screening dataset, and discuss the ethics of selecting a statistical analysis method. This case covers a broad range of topics including high throughput screening methods, drug discovery, cancer biology, statistical analysis selection, and research ethics. The wide range of topics allows instructors to customize the material to best suit their particular course which makes the case study very versatile.

This case can be implemented within one 50-minute lesson (for graduate students) or 1.5-2-hour lab (for undergraduate students). It is recommended that students have a basic understanding of biology and mathematics, but no advanced knowledge of pharmaceutical science, cancer biology, or statistics is required. Students do not have to have coding experience, as the activity is done in Microsoft Excel or Google Sheets. A pre-class reading assignment is provided and should be completed by students before the in-class activity to ensure there is enough time for the lecture and in-class data activity.

After a brief lecture from the instructor, students will be given a dataset generated from a high throughput drug screen and will be asked to analyze the data using two different methods: Z’ analysis and Z* analysis. Students will use this activity to determine the statistical method that best fits their data based on the experimental parameters. Students will then use the proper statistical method to determine the biological significance of their data by determining which drugs caused a change in biological activity. Students can then explore drug discovery methods by describing what they think would be the next steps once they have identified their lead drug molecules.

**Objectives**

The course learning outcomes relevant to the case study state that on successful completion of the course students should be able to:

- Define phenotypic cell-based screening and identify appropriate screening controls.
- Apply statistical modeling to a phenotypic screen to identify biologically meaningful results.
- Interpret the biological significance of a Z-value and a Z*-value.

**CLASSROOM MANAGEMENT**

This case can be taught in as little as one 50 minute class for graduate students and 1.5 hours for undergraduate students, with pre- and post-work as homework. The classroom time focuses on the synthesis and application of course material. Students should have laptops and each classroom should have wireless access points for use. Students should be instructed to bring their internet accessible device to class.

***Before Class***

Students are provided with a pre-class reading assignment that gives them background information on the two common high throughput screening methods in drug discovery: target-based and phenotypic-based screening. The reading uses dialog between a new graduate student and her mentors to introduce the specific screen being used to generate the data for the in-class activity. The reading provides the necessary cancer biology and quantitative polymerase chain reaction (qPCR) background, as well as some discussion on the two main types of statistical analysis used for high throughput datasets: Z’ analysis and Z* analysis (Zhang, 2011). Statistical analysis methods are dependent on the experimental parameters of the screen and this case uses real-world datasets to illustrate this point to students.

Instructors should provide students with the pre-class reading assignment at least one week before the in-class activity session. The reading assignment is quite dense, so it is recommended that students have ample time to read it over before coming to class. The pre-class reading assignment includes a few questions throughout the dialog that can be assigned to students as a pre-class assignment. Going over these questions in the in-class lecture may be a good way to assess student understanding of the reading assignment.

***In Class***

The in-class portion of the case should begin with a 20–30-minute lecture and questions section to ensure students have a grasp on the background material and biological significance of the data they are analyzing. The lecture should focus on the aspects of the case that pertain to the course, meaning the lecture material can be customized to fit best into each individual implementation course. The main topics of this case are high throughput screening methods, drug discovery, cancer biology (specifically telomere maintenance in cancer), statistical analysis selection, and research ethics.

This case provides students with a hands-on experience using datasets collected from a real drug discovery research lab. The data analysis activity portion of the implementation can range from 30-60 minutes. Students will be given a dataset and will use this data to understand why statistical analyses differ based on study parameters. The instructor will guide the class through two types of statistical analysis to demonstrate how screening techniques differ in how they are analyzed based on experimental methods. This can direct the class into a discussion about how selecting statistical analysis methods must be based on scientific reasoning, not just choosing which method produces desired results. By the end of the in-class activity, students should be able to discuss what their “hits” (drugs that gave a detectably different Z* score) mean in the context of cancer biology. If part of the course curriculum involves pharmaceutical science or drug discovery, this is a great way to have students discuss what the next experimental steps should be based on their results.

***Post-Class***

A second dataset can be assigned to the students as homework for them to analyze on their own. Students can be given written instructions on how to perform Z’ and Z* analysis in case they get stuck on their own. We have also provided homework questions to accompany the data analysis to help assess student understanding of their results. The homework gives students the opportunity to explore what their data means and to do some research on drug discovery methods to help them describe hypothetical experiments.

**BLOCKS OF ANALYSIS**

This case focuses on the following topics:

- Drug discovery and screening methods in pharmaceutical science
- Telomere maintenance in cancer (specifically, the alternative lengthening of telomeres pathway)
- Quantitative Polymerase Chain Reaction (qPCR) and C_T_ values
- Statistical analysis
- Ethical practice in statistical analysis

***Drug Discovery***

This case discusses drug discovery which is the first step of the drug development process (Figure 1). Once a target protein or a biomarker of a target pathway has been established by basic science, high throughput compound screening is done to identify compounds that inhibit the target protein or activity associated with disease (Strovel et al., 2016). Once compounds that appear to interfere with target activity (“hits”) have been identified, they are optimized to increase potency and decrease off target effects(Sun et al., 2022). This step can also be called “hit to lead optimization”. Once drug optimization is complete, the drug will undergo pre-clinical testing which is usually done in animal models. If the drug is found to be successful with low toxicity in animals it can be carried into human trials (phase I, II, and III). If the drug candidate can successfully pass all three clinical trial phases, it is very likely to be approved by the federal food and drug administration (FDA). The process from screening to approval is extremely long and the probability of success at each step is extremely low (Figure 1).


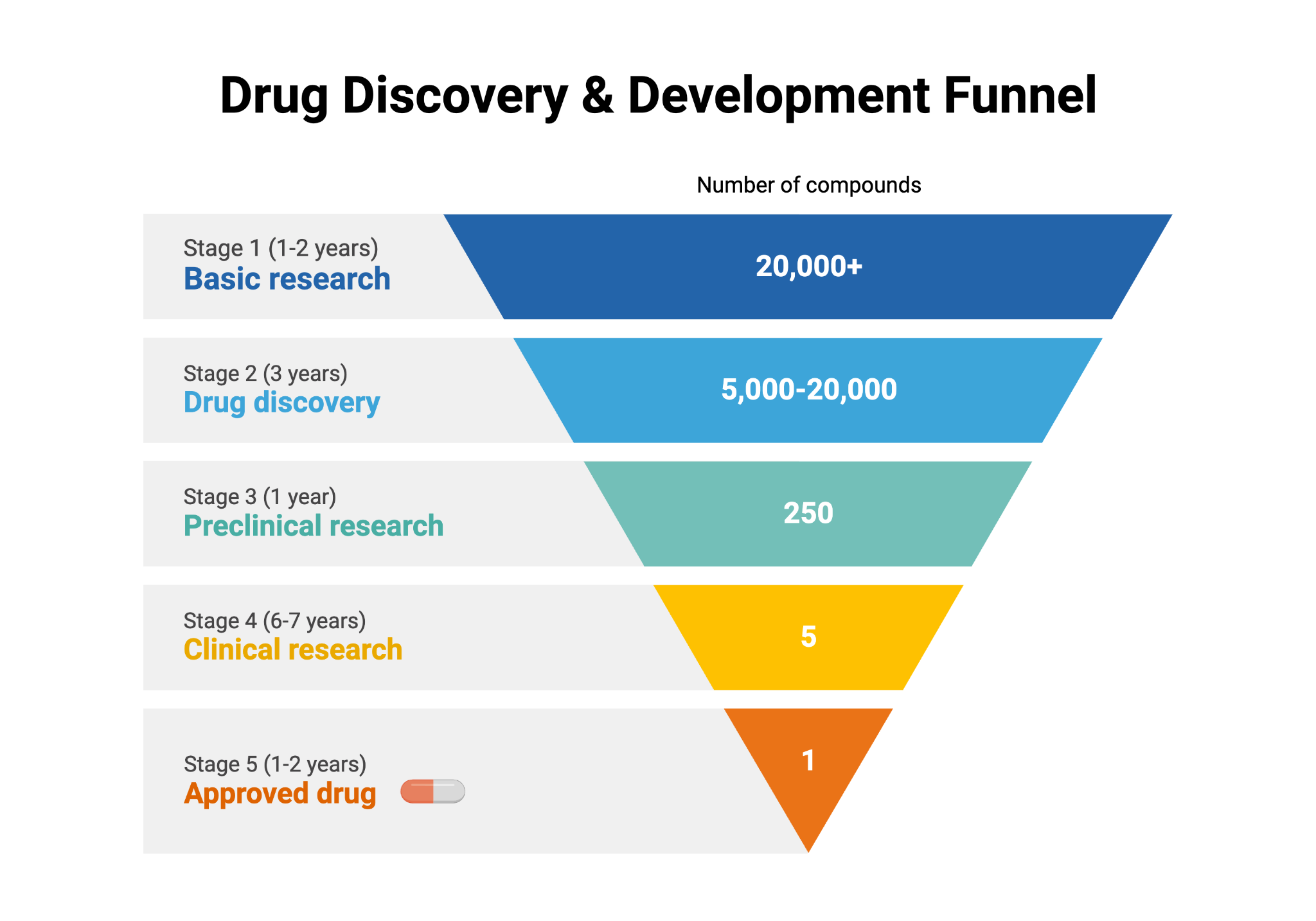


**Figure 1. The drug development funnel.** Image created with Biorender.com, generated from drug development steps described in literature (Sun et al., 2022).

This case mainly focuses on the high throughput screening aspects of drug discovery. The two main types of high throughput screens in pharmaceutical science are target-based and phenotypic-based screens. A brief description of these screening techniques as well as a comparison of the two can be found in the pre-class reading document along with references (Croston, 2017; Moffat et al., 2017). A basic understanding of drug discovery methodology and background is recommended (Strovel et al., 2016).

An understanding of screening controls is also recommended. A positive control should give you a high level of signal, while a negative control should have a low signal (i.e. noise) (Markossian et al., 2004). These controls are crucial for determining if your assay is functioning appropriately, and for measuring the effect of each drug in your screen. Ideally, you would like a large difference between the output of the positive and negative controls so that your signal to noise ratio is high. Large separation between controls also means you have a nice big screening window for detecting “hits” (drugs that have significant effect on target activity).

***Telomere Maintenance in Cancer***

Telomeres are long segments of DNA repeats found at the ends of chromosomes. They protect these ends from being recognized as double stranded breaks during DNA replication, allowing cell division to progress smoothly (Griffith et al., 1999). Because of the end-replication problem, telomeres shorten with each cell division until they are too short to successfully protect chromosome ends, signaling the cell to enter senescence (Hanahan and Weinberg, 2000). Cancer cells need to avoid senescence to achieve unlimited replicative potential and they do this by activating a telomere maintenance mechanism (TMM) (Sager, 1991). Since TMMs are crucial for the survival of cancer cells but not somatic cells, they pose as attractive cancer drug targets. There are two main TMMs activated by cancer: telomerase reactivation (Greider and Blackburn, 1989; Yu et al., 1990) and the alternative lengthening of telomeres pathway (ALT) (Cesare and Reddel, 2010). While telomerase reactivation is well understood, ALT is not which has resulted in a lack of ALT-specific cancer therapies. ALT cancers are highly aggressive and have poor patient outcomes, which emphasizes the need for better therapies for patients with ALT positive tumors. There is no known cellular target for ALT cancers, making a target based high throughput screen for identifying ALT-specific drugs impossible. There are, however, a handful of phenotypic biomarkers specific to ALT that can be used to develop a high throughput phenotypic screen for identify ALT inhibition (Henson et al., 2009). This case study describes a lab that has developed a high throughput phenotypic screen that uses an ALT specific DNA biomarker as a readout. This screen can help identify drugs that change the presence of this biomarker which likely means the drug is modulating ALT activity. Students will be able to identify the biological meaning of their results and use their data to describe what their “hits” mean in the context of cancer.

***Quantitative Polymerase Chain Reaction (qPCR) and C_T_ Values***

An overview of qPCR reactions can be found here: <https://www.sigmaaldrich.com/US/en/technical-documents/technical-article/genomics/qpcr/how-qpcr-works> (Sigma Aldrich). Each qPCR sample produces a C_T_ value which represents the PCR cycle at which the fluorescence readout of the sample reached a threshold determined by the qPCR software. The earlier the sample reached the threshold, the more target DNA (the gene the primers target) is present as it took less time for that sample to reach the threshold fluorescence. That means that a sample with a low C_T_ value has more target DNA present than a sample with a high C_T_ value.

***Statistical analysis (Z’ vs Z*)***

A Z’ value determines if the size of the screening window is large enough to accurately determine hits (Zhang et al., 1999). Z* analysis is used in instances where there is not a positive and negative set of controls, as is common in phenotypic screens (Zhang, 2011). Because phenotypic screens do not have the proper conditions for Z’ analysis, Z’ analysis doesn’t work for these datasets. The Z* metric is commonly used for non-normally distributed data and it normalizes the data to the median instead of the mean of the samples. The Z* metric uses the median of absolute deviations (MAD) in place of standard deviation. The pre-class reading assignment describes these metrics in more detail and includes some graphics to help illustrate the difference between the two metrics. We recommend writing the two equations on a board in the classroom, if possible, to refer to throughout the in-class data activity. It is helpful to have the equations to refer to as the class works through the analysis. The equations for both metrics are included in the pre-class reading and are repeated below (Zhang et al., 1999; Zhang, 2011):


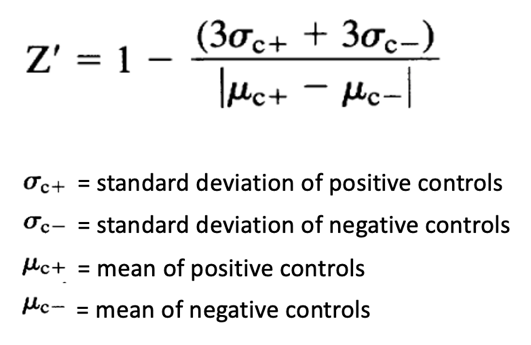


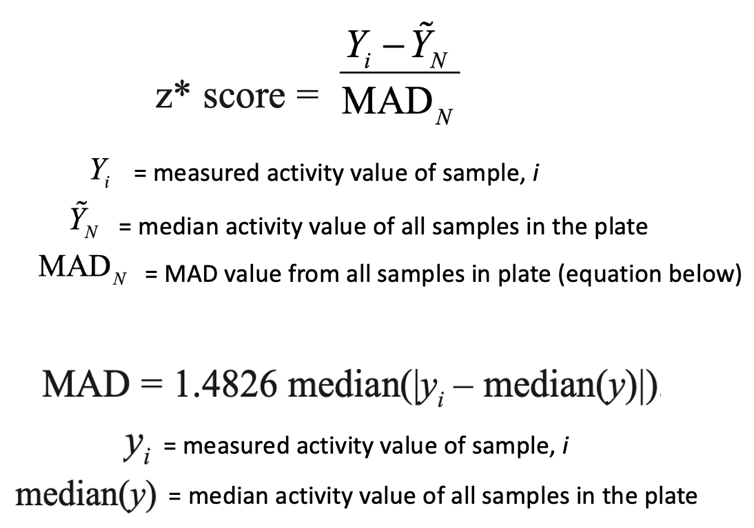


***Ethics behind statistical analysis***

This case involves students attempting to analyze a dataset using two different statistical methods and determining which analysis is best for their data. This activity should prompt students to think critically about the way to analyze and report data as it is very easy for scientists to ignore ethical responsibility when reporting findings. There is currently a problem with how scientists report or “spin” data to make conclusions that might not necessarily be proven by the data shown (Chiu et al., 2017). Discussing how to ethically choose a statistical method based on scientific reasoning is an important topic for young scientists to understand. Students could think critically about the potential consequences of irresponsible reporting of scientific findings and how data analysis methods fit into these situations. For instance, reporting a “hit” compound detected from an improper statistical method could cause other scientists to chase a lead compound for drug development that has no activity. Inaccurate detection of a hit compound would lead to a waste of time and funding. If a key part of the course curriculum is teaching students to understand the consequences of reporting data unethically or irresponsibly, this is a great discussion to include into the lesson plan.

**ASSESSMENT**

This case is made up of three parts: a pre-class assignment, an in-class activity, and a post-class homework. Before class, students are expected to read about the key topics using the provided materials and answer some short questions. Instructors have discretion in how they would like to grade the pre-class work (for completion or for credit). In class, students will work through some statistical analysis in groups or as a whole class, and then participate in a class discussion. Instructors may use the answer key to grade the statistics activity and their own rubrics to grade student participation in the class discussion. The main assessment is a short quiz given to the students at the beginning and end of class. This quiz is designed to measure student understanding of the three main learning objectives. Finally, students will analyze a new data set after class for homework. They will also be given extension questions and the instructor can grade the homework using the provided answer key.

**ANSWER KEY**

The answer to questions as well as completed in-class and homework data workbooks are found in separate documents available to instructors.

**REFERENCES**

Cesare, A. J., and Reddel, R. R. (2010). Alternative lengthening of telomeres: Models, mechanisms and implications. *Nat Rev Genet* 11, 319–330. doi: 10.1038/nrg2763.

Chiu, K., Grundy, Q., and Bero, L. (2017). ‘Spin’ in published biomedical literature: A methodological systematic review. *PLoS Biol* 15. doi: 10.1371/journal.pbio.2002173.

Croston, G. E. (2017). The utility of target-based discovery. *Expert Opin Drug Discov* 12, 427–429. doi: 10.1080/17460441.2017.1308351.

Greider, C. W., and Blackburn, E. H. (1989). A telomeric sequence in the RNA of Tetrahymena telomerase required for telomere repeat synthesis.

Griffith, J. D., Comeau, L., Rosenfield, S., Stansel, R. M., Bianchi, A., Moss, H., et al. (1999). mammalian telomeres end in a large duplex loop. *Cell* 97, 5032–514.

Hanahan, D., and Weinberg, R. A. (2000). The Hallmarks of Cancer Review evolve progressively from normalcy via a series of pre.

Henson, J. D., Cao, Y., Huschtscha, L. I., Chang, A. C., Au, A. Y. M., Pickett, H. A., et al. (2009). DNA C-circles are specific and quantifiable markers of alternative- lengthening-of-telomeres activity. *Nat Biotechnol* 27, 1181–1185. doi: 10.1038/nbt.1587.

Markossian, S., Grossman, A., and Brimacombe, K. (2004). *Assay Guidance Manual*. Eli Lily & Company and the National Center for Advancing Translational Sciences.

Moffat, J. G., Vincent, F., Lee, J. A., Eder, J., and Prunotto, M. (2017). Opportunities and challenges in phenotypic drug discovery: An industry perspective. *Nat Rev Drug Discov* 16, 531–543. doi: 10.1038/nrd.2017.111.

Sager, R. (1991). Senescence As a Mode of Tumor Suppression.

Sigma Aldrich. (2023). How qPCR Works. <https://www.sigmaaldrich.com/US/en/technical-documents/technical-article/genomics/qpcr/how-qpcr-works> [Accessed November 13, 2022].

Strovel, J., Sittampalam, S., Coussens, N. P., Hughes, M., Inglese, J., Kurtz, A., et al. (2016). Early Drug Discovery and Development Guidelines: For Academic Researchers, Collaborators, and Start-up Companies. Available at: https://www.ncbi.nlm.nih.gov/books/.

Sun, D., Gao, W., Hu, H., and Zhou, S. (2022). Why 90% of clinical drug development fails and how to improve it? *Acta Pharm Sin B* 12, 3049–3062. doi: 10.1016/j.apsb.2022.02.002.

Yu, G.-L., Bradley, J., Attardi, L., and Blackburn, E. (1990). In vivo alteration of telomere sequences and senescence caused by mutated RNAs. *Nature Publishing Group* 344.

Zhang, J.-H., Chung, T. D. Y., and Oldenburg, K. R. (1999). A Simple Statistical Parameter for Use in Evaluation and Validation of High Throughput Screening Assays. *J Biomol Screen* 4, 67–73. doi: 10.1177/108705719900400206.

Zhang, X. D. (2011). Illustration of SSMD, z score, SSMD*, z* score, and t statistic for hit selection in RNAi high-throughput screens. *J Biomol Screen* 16, 775–785. doi: 10.1177/1087057111405851.

**SUPPLEMENTAL MATERIAL**

1. Pre-class reading assignment and data analysis instructions (pre-class reading questions embedded)
2. Pre- and post-class short quiz
3. In-class dataset
4. Homework dataset, instructions, and extension questions and answer keys
