# Supplementary material for: Behind the screen: drug discovery using the big data of phenotypic analysis
Source: Front Educ (Lausanne). Author manuscript; Available in PMC 2024 Sep 5. (PMC11376653; doi:10.3389/feduc.2024.1342378)
Supplement: Table 10 [file NIHMS1969654-supplement-Table_10.docx]

Behind the Screen: Drug Discovery using the Big Data of Phenotypic Analysis

Prework Part I: A research student, Merry, in a pharmacology/drug discovery program is joining a lab group focused on developing high-throughput screens

Merry is a first-year graduate student who just finished doing her rotations in pharmacology and drug discovery labs - she was accepted by her first choice of labs! She had a meeting with her new PI Dr. Sam to discuss potential projects involving screening compounds to identify new therapeutic agents or applications. She’s really excited about identifying molecules that have potential as therapeutic agents in cancer patients. Merry’s advisor is most enthusiastic about an idea for a new type of screen - a phenotypic screen. As soon as she gets home, Merry looks up resources to make sure she understands exactly what her project might entail.

Merry knows from her program coursework that high throughput screening is a common technique used as the starting point for drug discovery campaigns. Through her research, Merry learned that the two main approaches used in high throughput screening for drug discovery are **target-based screens** and **phenotypic (cell-based) screens**.

In a **target-based screen**, a disease-specific protein is targeted with a library of small molecule inhibitors. Target-based screens can be as simple as searching for a compound that inhibits the activity of a purified enzyme in a tube. More reading on target-based screens can be found in the article referenced at the end of this document titled *The utility of target based discovery* (Croston, 2017). Target-based screens can be performed in assay plates that have 96-wells, 384-wells, or 1536-wells, allowing hundreds, thousands, and even millions of compounds to be screened. Since these types of screens involve a known disease-specific cellular component, target-based screens can identify hit compounds that may potentially be optimized to possess the most desirable profile in the target class (i.e. “best in class” drug).

**Phenotypic screens** are applied to diseases where there are no clear therapeutic targets (target agnostic approach). For instance, there are many health conditions, such as certain aggressive cancer types, that are poorly understood at the molecular level. It is not possible to perform a target-based screen when no specific cellular proteins or pathways are known to drive the disease. Even if a specific target is unknown, however, many of these diseases have characteristic cellular, biochemical, or molecular changes (also called “biomarkers”) that can be measured. Phenotypic screens evaluate changes in the presence of a disease-specific cellular biomarker. Some examples of cellular biomarkers include irregular expression of a gene, buildup of DNA structures in the nucleus, or accumulation of mutated protein. More information on phenotypic screening can be found in the review article listed at the end of this document (Moffat et al., 2017). Phenotypic screens can be performed on cells grown on plates or even in whole organisms such as fruit flies. Hit compounds will alter the biomarker of interest. Since phenotypic screens are performed on systems where little is known about the mechanisms causing disease, hit compounds can be used to identify new therapeutic targets and potentially be optimized to “first-in-class” drugs.

To help organize her research, Merry generated a chart comparing the two techniques to help her understand the pros and cons of each screening approach (Swinney, 2013):

| Target based (in vitro) screen | Phenotypic (cell based) screen |
| --- | --- |
| Can lead to “Best-in-class” drugs | Can lead to “First-in-class” drugs |
| Simple readout, high throughput | Time consuming, high throughput not always possible |
| Target identified *a priori* | Target deconvolution can be challenging |
| High and low controls simple to define | Not always possible to have a high and low control |
| Simple statistics to define assay window and hits | Statistical approaches must be customized to the assay to account for biological variability and lack of simple assay controls |
| Hits may not have activity in cells | Hit activity is cell-based |

LO1: Define phenotypic cell-based screening & identify appropriate screening controls

1. **In your own words, describe a phenotypic screen? How is it different from a target-based screen?**
2. **What controls do you think would be necessary in a phenotypic screen?**

**Pre-Work Part II: Screening Statistics**

*Statistical explanation of when and how to apply Z analysis. Practical pro/con of their use.*

Merry noted that it is important in high throughput screening to ensure that you have an acceptable screening window, which is determined by defining the separation between positive and negative controls and allows for accurate detection of hit compounds. Assessing a screening window involves statistical analysis and proper selection of controls. Merry is not very experienced in statistical analysis so she asks the post-doc in her lab named Harold for a brief explanation of screening statistics.

“Hi Harold, I’ve done some reading up on target-based vs phenotypic screens and I noticed that there were some differences in how hits are identified in each screen type. Statistics isn’t quite my strong suit yet, do you mind explaining to me the different types of metrics used to assess screening data?”

Harold happily replied,”Of course! It is normal to be confused at first, I’d be happy to clear up some of the confusion!”

“When you set up a screening experiment on a plate, let’s use a 384-well plate for example, you need to set aside a certain set of wells to put control samples in. These controls help determine the size of your screening window and if your screening window is large enough to accurately detect hits. A common metric used to assess the size and acceptability of a screening window is a Z’ score, which uses the distribution of the controls to determine the size of the screening window (Zhang et al., 1999). Usually you have a set of controls that produce a high readout, called positive controls, and a set that produces a low readout, negative controls. These controls usually result in a normal distribution meaning if you plot them out they result in two bell shaped curves that look something like this:”

“Most of the control values will fall along the black line down the middle of the bell curve, and the blue outline of the ‘bell’ represents 3 standard deviations from the average control readout value. Now, if a compound treated sample readout falls within the dotted lines of the controls, that means the readout is not statistically significantly different from the controls meaning that compound is not a hit. If a treated sample falls beyond the three standard deviation line of the negative controls, that sample has generated a significantly different readout, meaning the compound is a hit. Since we are looking for samples that fall beyond 3 standard deviations of the negative controls, we need to make sure that the separation band between the two control sets is large enough to actually detect hits. For instance if we had a set of control curves that looked like this:


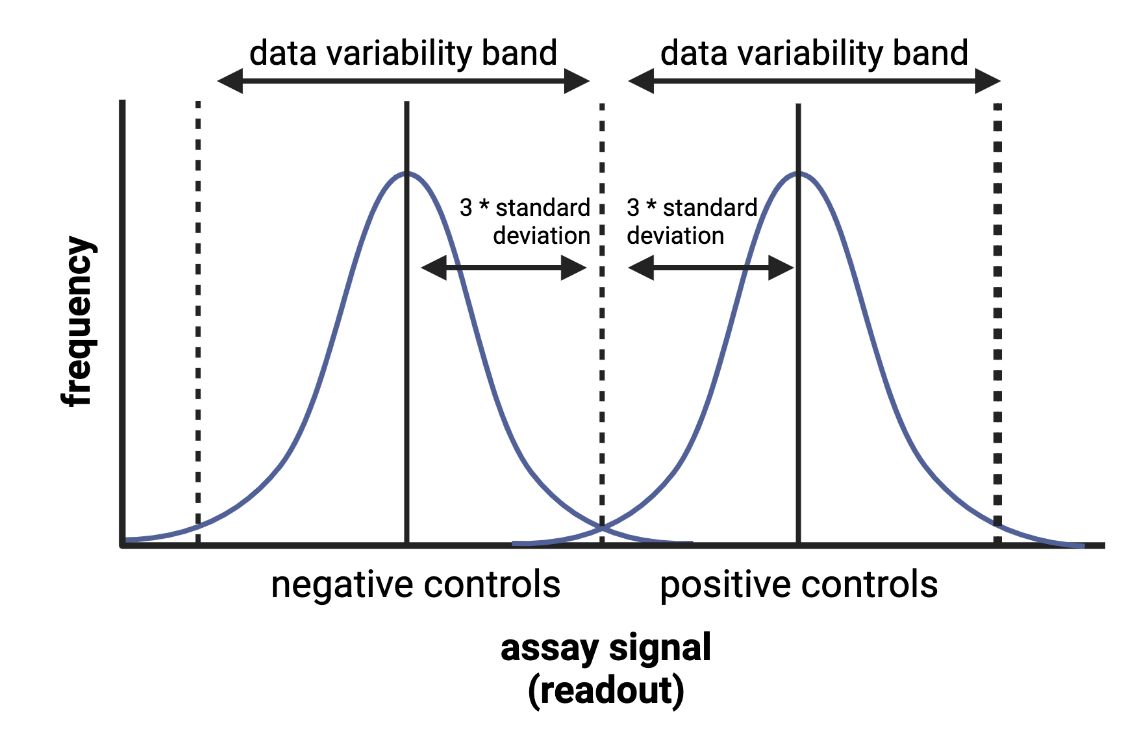


“There’s no separation band between the two control curves to detect if we have a hit sample. We want our curves to look like this so that we have an acceptable screening window for hit identification:


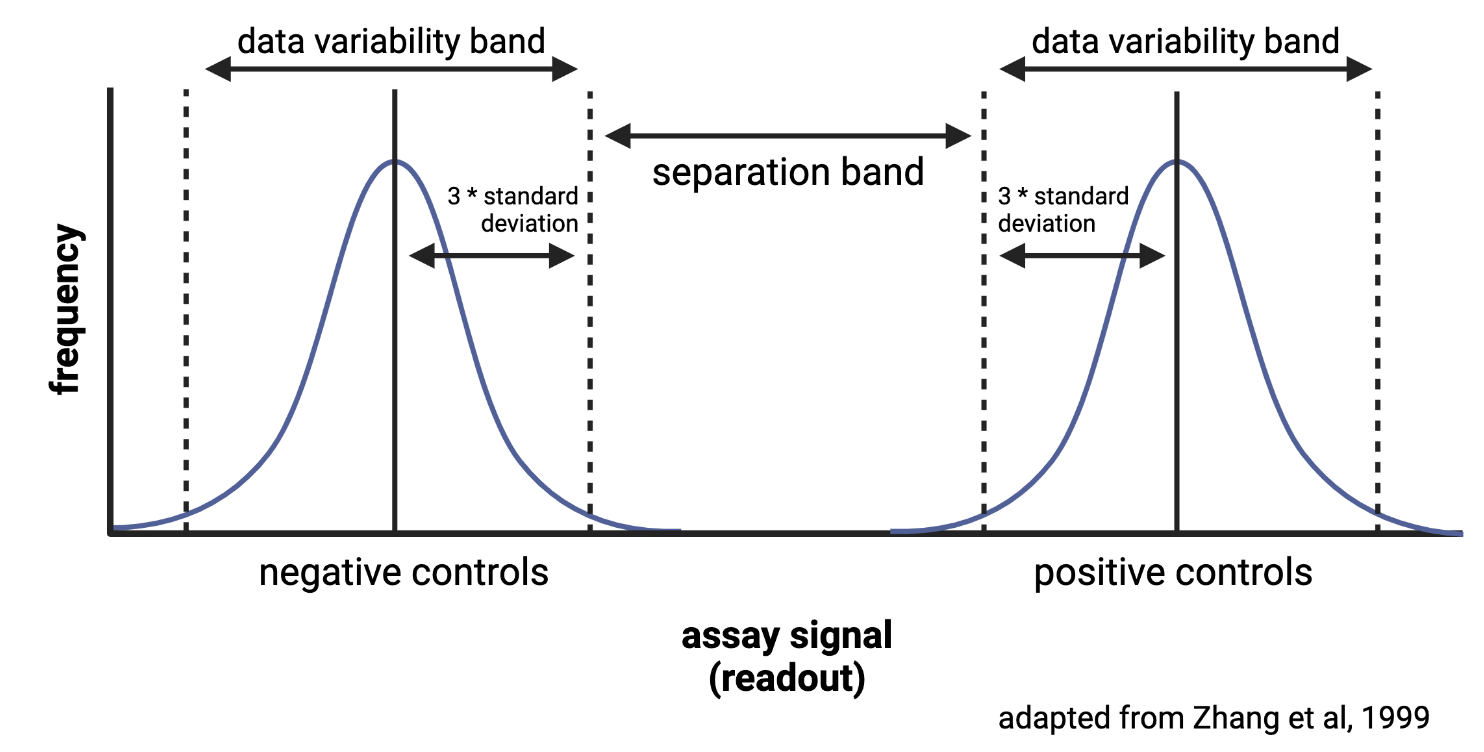


“We determine the size of the separation band statistically by using the metric called Z’ score which uses the following equation (Zhang et al., 1999):


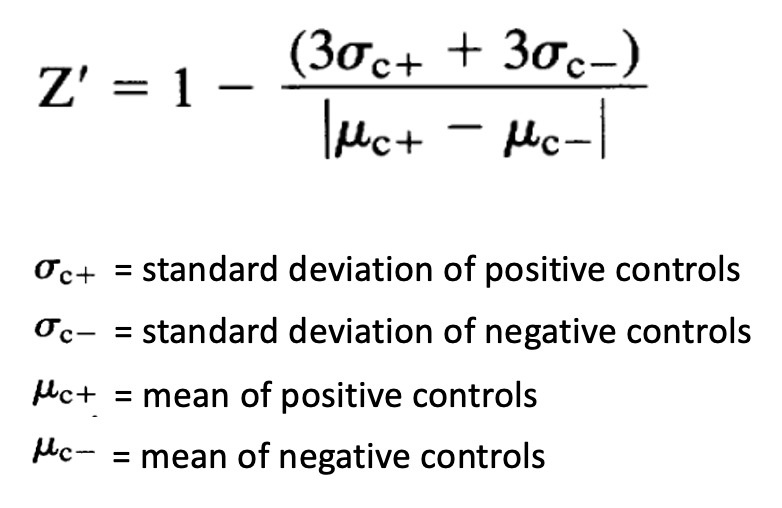


If the Z’ score of an experiment falls between 0.5 and 1, the screening window is deemed acceptable and hits can be determined from the treatment sample group. If the Z’ score of an experiment falls below 0.5, the separation band between the positive and negative controls is not large enough to accurately detect hits. So, Z’ is great for when you have controls that are normally distributed, like the curves I’ve drawn above. While controls in target-based screens produce data with a normal distribution, phenotypic screen controls tend to be more complicated. Since phenotypic screens assess the behavior of complex cellular systems, the controls do not produce the normally distributed bell curves like the ones above. They look more like this:


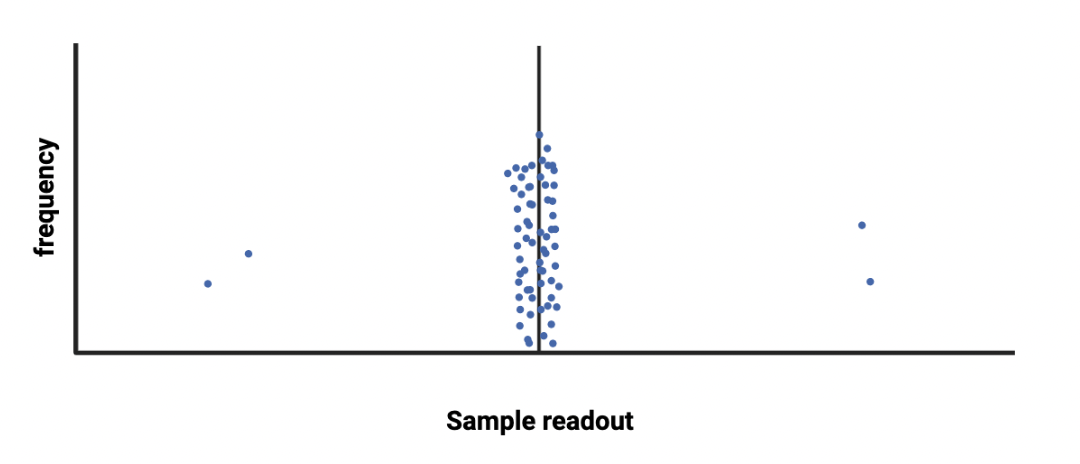


“You can see the control sample readouts all cluster together rather than form a normally distributed bell curve (represented by the faded dashed blue lines). This makes the controls incompatible for the Z’ metric which requires normally distributed controls. However, in a phenotypic screen it is not always possible to have positive and negative controls. You can see that most of our samples fall around a median readout value and samples that give us very low or very high readouts (hits) look like outliers to the rest of the data points. So, while we can see from this plot there are hit samples, the data is not distributed in a way that is conducive to a Z’ score analysis and will produce a Z’ score lower than 0.5, because those outliers cause a high standard deviation. However, because the majority of samples have similar readout values, a readout that deviates from the control readouts, AKA a hit, would look like an outlier of the data and we can use a different metric to detect outliers by using the median instead of the mean. This alternative way to analyze high throughput screening data is the median-based metric called Z*(Zhang, 2011), which determines hits based on how much they deviate from the median readout value:


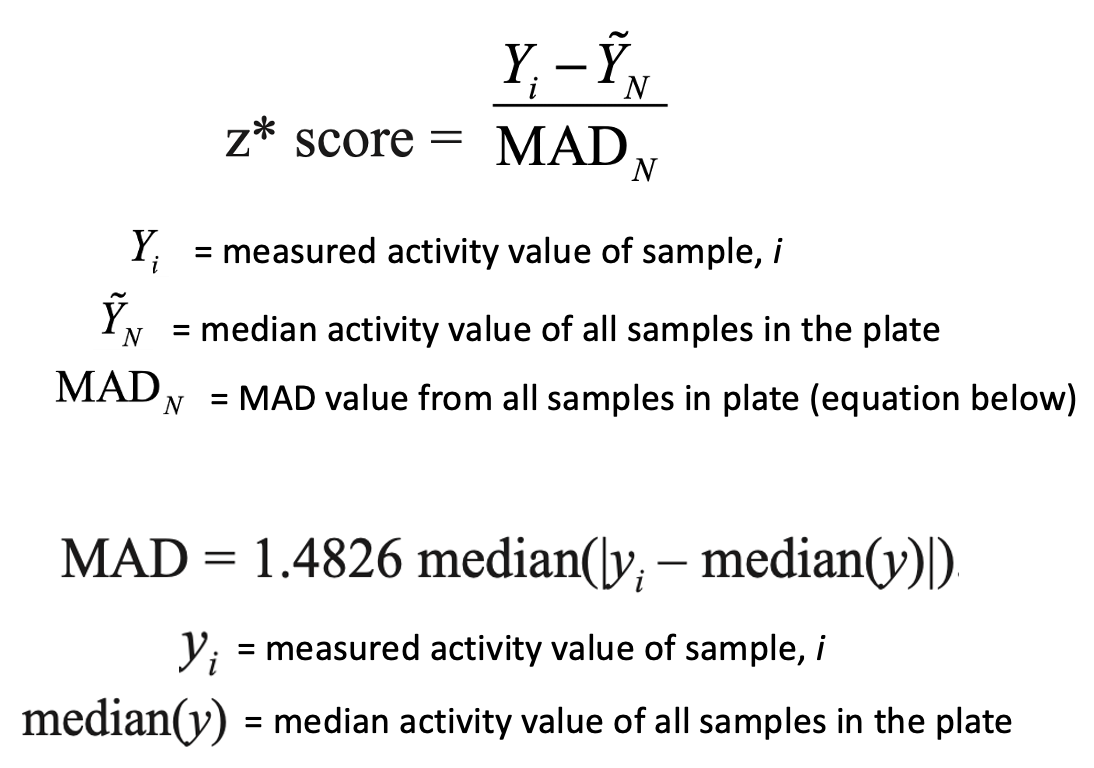


“When we do z* analysis our data looks like a scatter plot and we plot a line at + and - 3MAD as guidelines for determining hits:


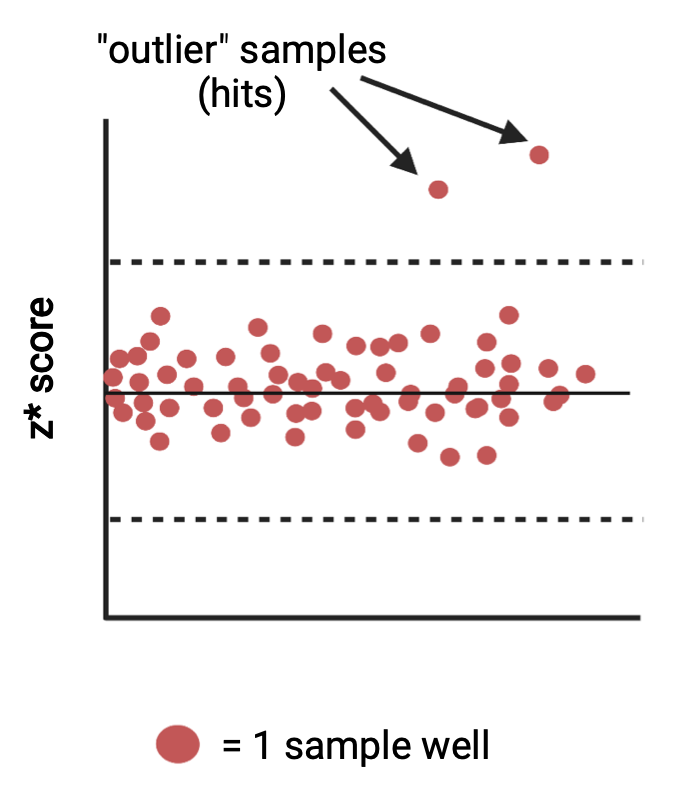


“So, most of the samples and controls fall within the + or - 3MAD lines and any sample that produces a readout outside of those lines is considered a hit since it is statistically significantly different from the median readout value as determined by Z* analysis.”

Merry now has a great understanding of how to analyze screening data using Z’ and Z* analysis and is eager to learn how to set up these types of experiments. “So, how many control wells do you need to have on the plate to get an accurate screen?”

“Great question,” Harold replied. “Control well number and layout actually depend on what type of analysis you plan to use for your experiment. For Z’ analysis it is common practice to plate controls on the outer edges of the assay plate like so:


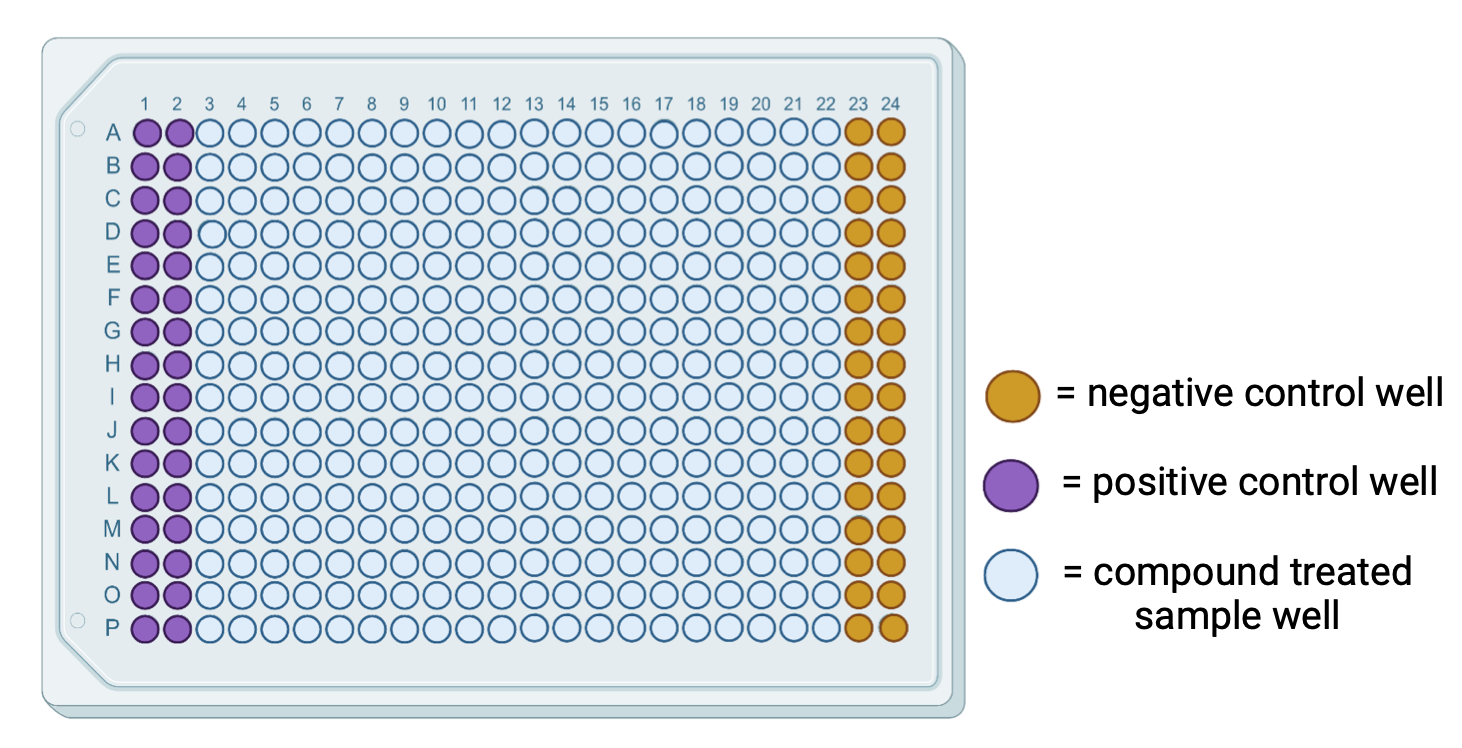


“We want a large set of control wells to ensure that we get a robust set of bell curves which will help achieve an acceptable Z’ score. If an acceptable Z’ score is obtained, a Z’ value can be calculated for each data point and the Z’ values can be plotted out like so:


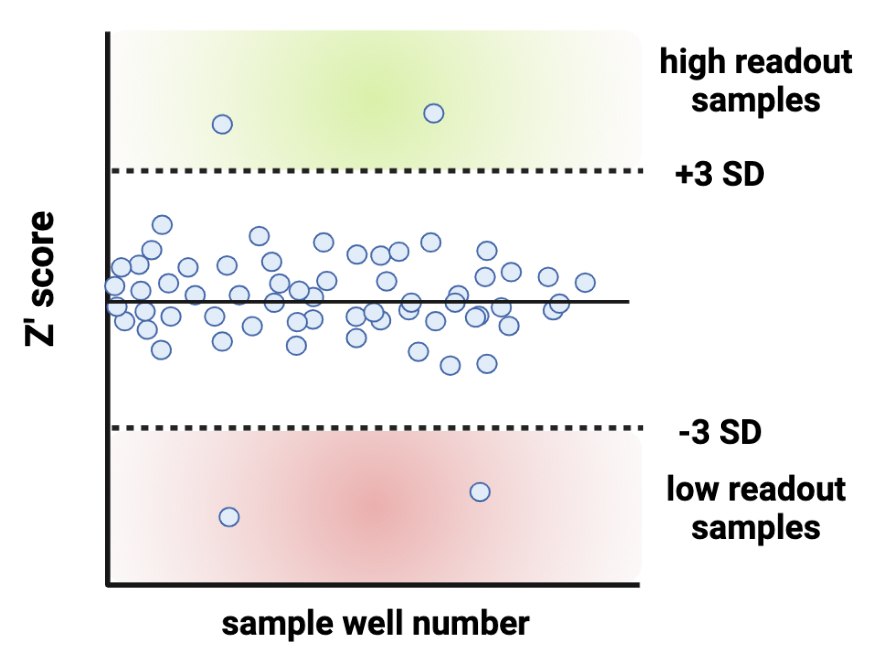


“Any sample that falls above or below the 3 x standard deviation value would be considered a hit. On the other hand, in Z* analysis hits are identified as outliers from the rest of the data and since hits only make up about 0.5-1% of the samples in a screening library, most of the treated samples will produce readout values that resemble positive controls. Because of that, the treated samples can actually act as their own positive controls, meaning you only need to plate out negative controls for Z*analysis. When we plate out Z* experiments they tend to look more like this:


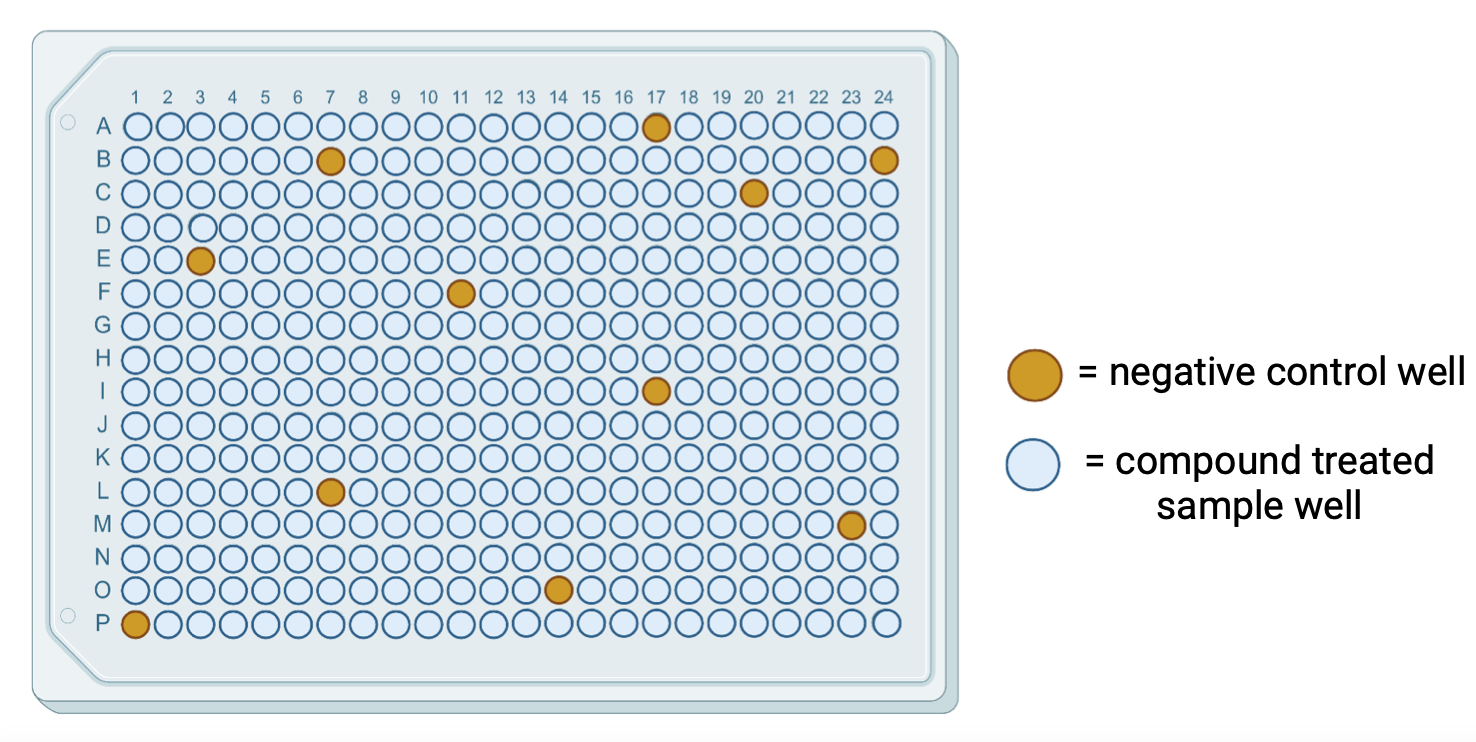


“We scatter the negative controls randomly across the plate to check for plating bias because technical errors during plating can occur and can cause variability in our results. We calculate a Z* score for each sample including the negative control samples and plot them like so:


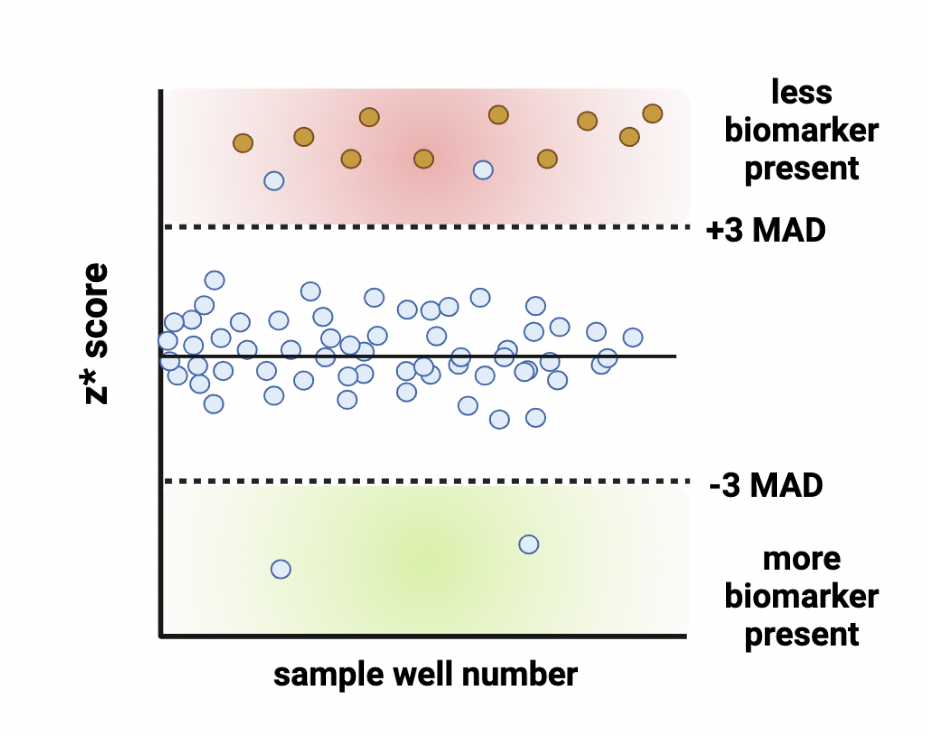


“This looks similar to the Z’ graph except instead of hits falling above or below 3 x standard deviation, they fall above or below 3 x MAD. You may also notice that samples that give a Z* score above 3 x MAD has less of the target biomarker present which suggests the compound given to this sample may be inhibiting our target pathway. On the other hand, a sample with a Z* score that falls below 3 x MAD has a higher amount of the biomarker present which implies that compound treatment in this sample may have enhanced our target pathway. We expect our negative control samples to all fall above the +3MAD line since these samples have no biomarker present and our assay should be able to detect that absence of biomarker as a large deviation from the median.”

“I see,” Merry replied. “I think I’m getting the hang of screening now!” Merry was now eager to meet with her PI to discuss what her specific screening project would entail.

LO1: Define phenotypic cell-based screening & identify appropriate screening controls

LO2: Apply statistical modeling to a phenotypic screen to identify biologically meaningful results

1. Now that you are more familiar with how Merry will analyze the data, what controls does she need? Ensure that your answer discusses positive and negative controls.
2. Merry is now in the data analysis phase. Does Merry need to do both the Z’ and Z* analysis for her phenotypic assay?

**Pre-Work Part III: Cancer- Specific Phenotypic Assay**

Merry met with her PI, Dr. Sam, to discuss her role in the project. “Hi Merry. I know that you are anxious to get started. Before you do, I wanted to chat with you about the cancer-specific pathway that we are studying. As you know, an important hallmark of cancer is unlimited proliferative potential, meaning cancer cells are able to override cellular signals that limit cell division in somatic cells (Hanahan and Weinberg, 2000). One of the cellular signals that induces senescence is telomere length. Telomeres act as the “end caps” of chromosomes to ensure DNA replication and cell division go smoothly. When telomeric DNA segments become too short, the cell is signaled to enter senescence and can no longer divide (Griffith et al., 1999). Cancer cells need to figure out a way to lengthen these telomeric segments to avoid senescence and they do this by activating a telomere maintenance mechanism (TMM) (Sager, 1991). There are two TMMs utilized by cancer cells: telomerase reactivation and alternative lengthening of telomeres (ALT). Of the two TMMs, there is much less known about the ALT pathway (Cesare and Reddel, 2010). ALT positive tumors are usually very aggressive and have poor patient outcomes, so there is a great clinical need for an ALT-specific therapy but no ALT inhibitors have been developed. There are currently no ALT inhibitors in development because no ALT-specific cellular drug targets have been identified. Because of this fact, target-based high throughput screens can not be used for ALT-specific drug discovery at this time. Our lab has developed a phenotypic screen using a biomarker that is specifically expressed in ALT cells. When cancer cells become ALT-positive, they produce circles of DNA called C-circles in their nuclei that we can identify using the polymerase chain reaction (PCR)(Henson et al., 2009). Our hypothesis is that the C-circle biomarker will decrease in the presence of “hit” compounds. We expect this phenotypic assay to identify first-in-class compounds that may become drugs for treating ALT positive cancers. We will initially screen a 600-compound small molecule inhibitor library in both ALT-positive and ALT-negative cancer cells. We are interested in compounds that decrease C-circle levels in ALT-positive cells, but have no effect on the ALT-negative cells (which do not have the C-circle biomarker).

“That sounds wonderful. I have always wanted to do cancer research that can benefit patients with aggressive tumors. What type of techniques will I need to know?”

“Great question Merry. We routinely work with the quantitative polymerase chain reaction, or qPCR. You will need to be familiar with Ct values and Z-analysis. We have a great team who will get you started.”

Merry is excited to begin working on the drug screening. The post-doc, Harold, starts chatting as he gives her a tour of the lab. “Hi Merry, glad to have you with us. We are preparing to run a qPCR analysis of the first 300 compounds of our screen, would you like to assist?” Merry, realizing that she has much to learn, replies “Sure!”. Harold recognizes her hesitation and offers, “I’ll give you a brief overview of qPCR to catch you up!”

“qPCR (quantitative Polymerase Chain reaction) allows us to measure target gene amplification in real time by monitoring fluorescence dye signals. Each reaction contains sample DNA, single stranded DNA primers specific for the target gene, dNTPs, magnesium, DNA polymerase, and a fluorescent dye. Within each cycle of the qPCR reaction, sample DNA is denatured or melted, meaning the two complementary strands come apart to form single stranded DNA. If the target gene is present in the sample, the single stranded DNA primers that are complementary to the gene of interest will connect with the sample DNA to form a short double stranded DNA segment. The DNA polymerase will then start building the complementary strand using the dNTPs in the reaction well and this will create a new copy of the target DNA. The fluorescence marker binds to newly synthesized double stranded DNA which then gives a fluorescence readout for that cycle. This amplification occurs every cycle and the fluorescence signal of each cycle is plotted over time to give an amplification curve for each sample. We use qPCR software to determine a fluorescence value called a threshold value and we measure how much of our target gene is in each sample by determining what cycle number of the qPCR reaction target amplification reached that threshold value. We call this value the Ct value (cycle, C, at which the amplification curve reached the threshold, t). You need to remember that when it comes to Ct values, smaller is always better. “Why is this so?” Merry asked. Harold explained that a sample that reached the threshold at an early cycle has more target DNA present relative to a sample that reached the threshold at a later cycle. In other words, “the lower the Ct value, the more target DNA present in a sample.” Merry asked what would happen if the Ct value gets too high. Harold indicated that it will be quite difficult to determine real signals from background noise if this happens. He further explained that this won’t happen in their lab because there is already an established Ct cutoff and any data above the Ct cutoff value will not be used.

Harold also gave Merry a link to a short video to help visualize qPCR reactions: <https://www.sigmaaldrich.com/US/en/technical-documents/technical-article/genomics/qpcr/how-qpcr-works> (Sigma Aldrich)

“So Harold,” Merry started,”how does our qPCR data help us determine C-circle level in our cells?”

“Great question Merry! We use two different sets of primers in our assay. The first set we use targets and amplifies telomeric DNA. These primers amplify the C-circles in our samples since the C-circles are made from telomeric DNA. So our telomeric DNA primers amplify our target gene. We need to normalize our telomeric DNA expression to background gene expression in order to accurately detect samples that have higher target gene expression. We do this by running a second qPCR experiment using a primer set that targets RPLP0, a housekeeping gene that is regularly expressed in all cells. So, for each sample we get a Ct value for telomeric DNA expression and a Ct value for RPLP0 expression. We then normalize the telomeric DNA expression of a sample to the RPLP0 expression by doing what we call a ΔCt (delta Ct) analysis. This essentially is done by dividing the telomeric DNA expression by RPLP0 expression to give us a fold change difference in expression. Since qPCR amplification curves are logarithmic curves, our Ct values are in log form. That means if we want to divide telomeric DNA by RPLP0 we subtract the RPLP0 Ct from the telomeric DNA Ct. This gives us our delta Ct value, which is in log form. Our statistical analysis requires our data to be in the log form so we leave it like that, however you can calculate the fold change of telomeric DNA expression compared to RPLP0 expression by linearizing the delta Ct value.”


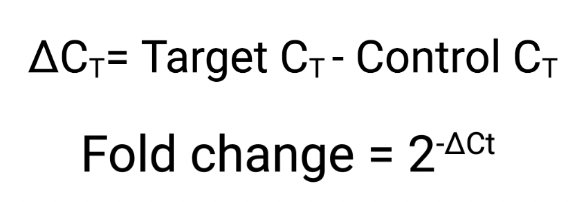


**POST-READING QUESTIONS**

1. Merry is trying to find a cancer drug, what is she measuring in her phenotypic assay? (1-2 sentences)

1. What would be different about the screen if it was a target-based screen?

3. Rate your level of confidence using the scale below to:

LO1: Define phenotypic cell-based screening & identify appropriate screening controls

1. Very Confident - I can do this easily
2. Confident - I may need to check resources but could figure it out
3. Somewhat Confident- I may need to ask for help but could figure it out
4. Poorly Confident - Even with resources and help it would still be vague
5. Not Confident - I can’t do it

LO2: Apply statistical modeling to a phenotypic screen to identify biologically meaningful results

1. Very Confident - I can do this easily
2. Confident - I may need to check resources but could figure it out
3. Somewhat Confident- I may need to ask for help but could figure it out
4. Poorly Confident - Even with resources and help it would still be vague
5. Not Confident - I can’t do it

LO3: Interpret the biological significance of a z-value.

1. Very Confident - I can do this easily
2. Confident - I may need to check resources but could figure it out
3. Somewhat Confident- I may need to ask for help but could figure it out
4. Poorly Confident - Even with resources and help it would still be vague
5. Not Confident - I can’t do it

**REFERENCES**

Cesare, A. J., and Reddel, R. R. (2010). Alternative lengthening of telomeres: Models, mechanisms and implications. *Nat Rev Genet* 11, 319–330. doi: 10.1038/nrg2763.

Croston, G. E. (2017). The utility of target-based discovery. *Expert Opin Drug Discov* 12, 427–429. doi: 10.1080/17460441.2017.1308351.

Griffith, J. D., Comeau, L., Rosenfield, S., Stansel, R. M., Bianchi, A., Moss, H., et al. (1999). mammalian telomeres end in a large duplex loop. *Cell* 97, 5032–514.

Hanahan, D., and Weinberg, R. A. (2000). The Hallmarks of Cancer Review evolve progressively from normalcy via a series of pre.

Henson, J. D., Cao, Y., Huschtscha, L. I., Chang, A. C., Au, A. Y. M., Pickett, H. A., et al. (2009). DNA C-circles are specific and quantifiable markers of alternative- lengthening-of-telomeres activity. *Nat Biotechnol* 27, 1181–1185. doi: 10.1038/nbt.1587.

Moffat, J. G., Vincent, F., Lee, J. A., Eder, J., and Prunotto, M. (2017). Opportunities and challenges in phenotypic drug discovery: An industry perspective. *Nat Rev Drug Discov* 16, 531–543. doi: 10.1038/nrd.2017.111.

Sager, R. (1991). Senescence As a Mode of Tumor Suppression.

Sigma Aldrich. (2023). How qPCR Works. <https://www.sigmaaldrich.com/US/en/technical-documents/technical-article/genomics/qpcr/how-qpcr-works> [Accessed November 13, 2022].

Swinney, D. C. (2013). Phenotypic vs. Target-based drug discovery for first-in-class medicines. *Clin Pharmacol Ther* 93, 299–301. doi: 10.1038/clpt.2012.236.

Zhang, J.-H., Chung, T. D. Y., and Oldenburg, K. R. (1999). A Simple Statistical Parameter for Use in Evaluation and Validation of High Throughput Screening Assays. *J Biomol Screen* 4, 67–73. doi: 10.1177/108705719900400206.

Zhang, X. D. (2011). Illustration of SSMD, z score, SSMD*, z* score, and t statistic for hit selection in RNAi high-throughput screens. *J Biomol Screen* 16, 775–785. doi: 10.1177/1087057111405851.

Figures in text created with BioRender.com.

**IN CLASS**

Following a successful qPCR run, Harold hands Merry a thumbdrive with the data. They ran two plates, one with controls set up for a Z’ analysis and one set up for a z* analysis. Merry opens the excel file ([DATA PROVIDED](https://docs.google.com/spreadsheets/d/1-v5CO6cWbxKHf2WUgCnP0j5JC0Ns8SRt/edit?usp=sharing&ouid=115355257413921971629&rtpof=true&sd=true)). She is prepared to analyze each data set.

With a solid understanding of target based screening and Z-analysis, Merry is ready to process her data.

**In Class Part II: Analyzing the data!**

Now it’s time for Merry to get into the data analysis in google sheets. ***Make a copy of the data that Harold gave Merry in your own google drive.***


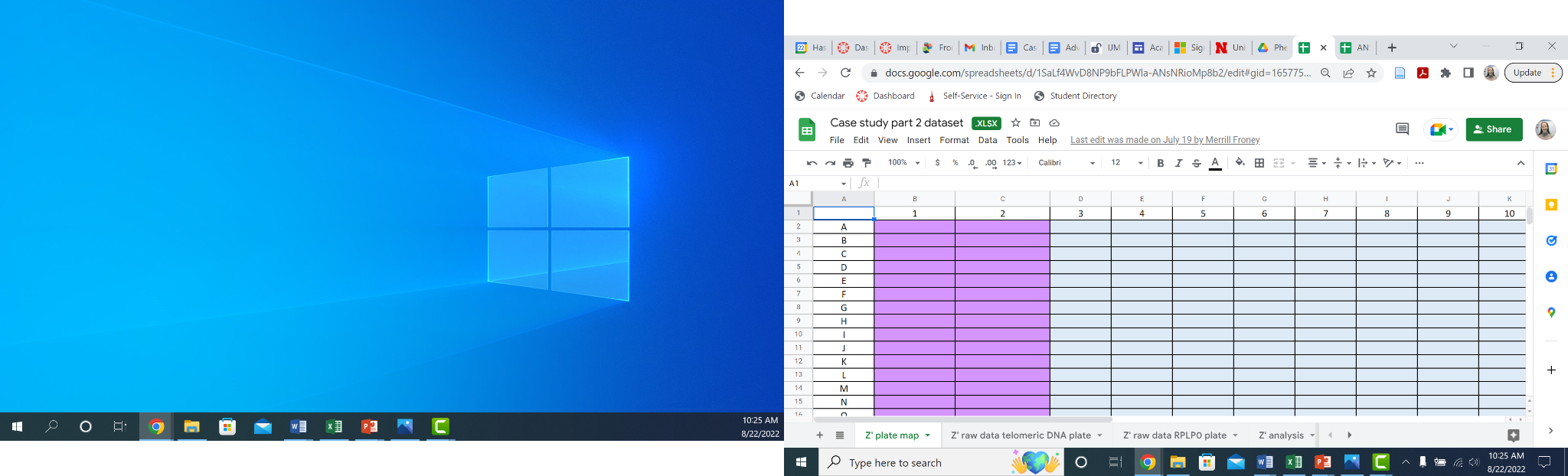


This file has multiple sheets that you can access by clicking on the sheet you would like to see. You can scroll through the sheets by pressing the right arrow on the bar with the sheet names. Note that since we have 384 samples in each plate, we needed to run two qPCR experiments: one for each primer set. The plate with primers that target telomeric DNA was run first and the plate with RPLP0 primers was run second.

1. **Z’ plate map**: This map is for your reference so you can visualize how the experiment was set up for Z’ analysis.
2. **Z’ raw data telomeric plate**: This is what qPCR data looks like when taken directly from the software. The telomeric DNA Ct value for each sample is provided in the column labeled “CT”. You may need to scroll down a little in the excel sheet as there is instrument label information that takes up the first 37 rows of the file.
3. **Z’ raw data RPLP0 plate**: This is just like the previous sheet, but it provides the qPCR data from the RPLP0 primer plate (our normalization control gene). Again, the Ct values are listed in the column labeled “CT”.
4. **Z’ analysis**: This is where the data from the two raw data files will be compiled and organized to make the Z’ calculations more efficient. The first column is the sample name, the second column is the Ct value of each sample from the telomeric DNA qPCR, and the third is the Ct value of each sample from the RPLP0 qPCR.

Now it is time to begin your analysis on the **Z’ analysis sheet**. Start by calculating the delta Ct for each sample in the experiment:

1. The fourth column should be empty. Label that column “delta Ct”.
2. In the first cell of that column (cell 2D), type “=”, then select the telomeric Ct for that sample (cell 2B), type “-”, and select the RPLP0 Ct (cell 2C). This value should be the telomeric Ct minus the RPLP0 Ct, AKA the delta Ct for that sample.
3. Hover your cursor over the bottom right corner of cell 2D until a black cross appears. Click on the corner with the cross cursor and drag the cross down the column until column D for all samples has been highlighted. Release the cursor. This should have copied the equation from cell 2D all the way down and each cell in column D should represent the delta Ct for each sample.

Now you are ready to calculate the Z’ score using the control delta Ct values:

1. First, calculate the average delta Ct of the untreated U2-OS (ALT+) cells. Do this by selecting cell G9 and typing “=average” and select the function called “AVERAGE”.
2. Select the delta Ct values for the U2-OS controls (samples in cells colored purple). Since the controls are spread out along the column, you will need to select the first two then type a comma (,), select the next two, and continue this pattern until all control sample delta Ct values have been included.
3. Calculate the standard deviation of the U2-OS controls by selecting cell G12 and typing “=stdev” and select the standard deviation function from the equation dropdown menu that pops up. Then select all the U2-OS control delta Ct values in the same way that you selected the values for the average calculation.
4. The Z’ equation uses 3 times the standard deviation to determine the screening window. In cell H12 type “=” followed by “3*” then select cell G12. This should calculate 3 times the standard deviation.
5. The same metrics need to be calculated for the MG-63 (ALT-) cell controls which are represented as yellow colored cells. Use cell G18 to calculate the MG-63 average delta Ct in the same manner that you used to calculate the average for the U2-OS controls.
6. Use cell G21 to calculate the MG-63 delta Ct standard deviation and cell H21 to calculate 3 times the standard deviation.
7. Next, calculate the fraction portion of the Z’ equation. The equation has been repeated below for convenience.


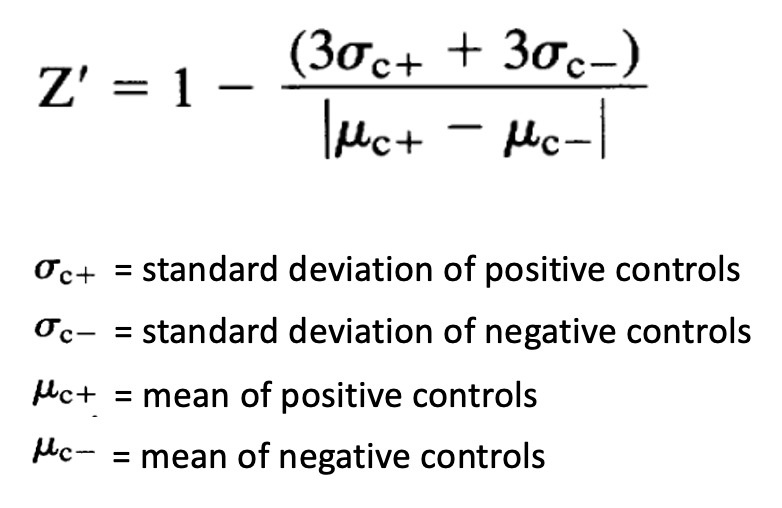


1. In cell G25 type “=(” then select cell H12, type “+”, select cell H21, then type “)”. In the same cell continue the equation by typing “/(abs” and select the absolute value equation from the dropdown. Then select cell G9, type “-”, select cell G18, and type “))”. So in full, cell G25 should read “=(H12+H21)/(ABS(G9-G18))”. This will give the fraction of the Z’ value.
2. To complete the equation select cell G28 and type “=1-” and select cell G25. This should give you the Z’ score for this experiment.

**Z Score Calculations**

|  | Average | SD | 3XSD |
| --- | --- | --- | --- |
| Untreated |  |  |  |
| Negative Control |  |  |  |

Z score _____________ In one sentence, what does this mean about the phenotypic screen results?

**
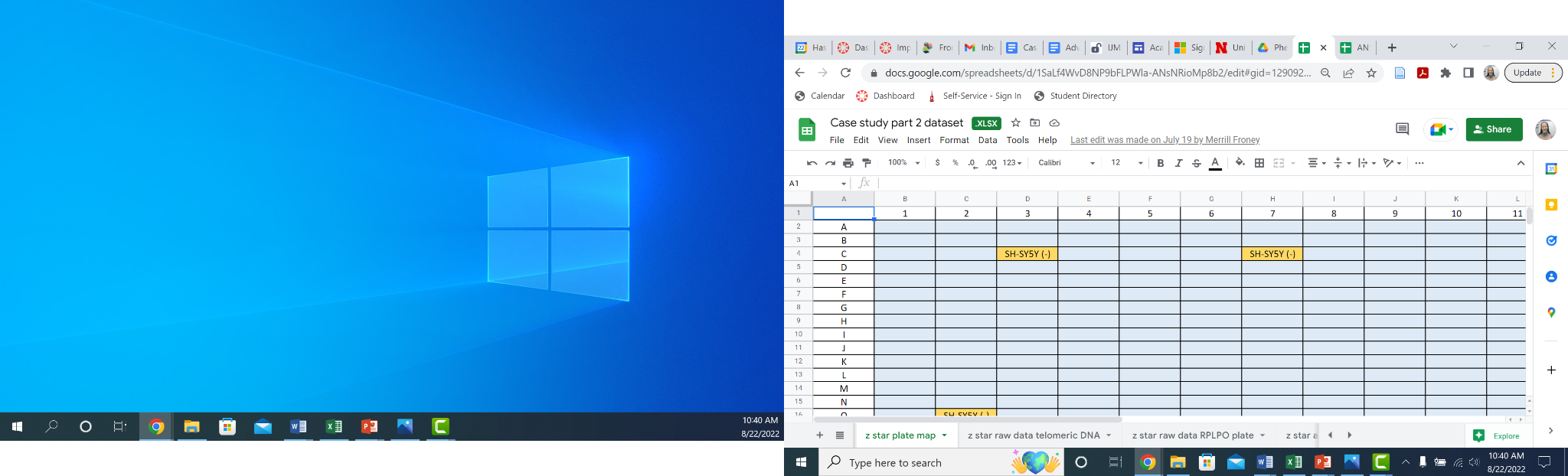
**

Now you will need to scroll through the sheets by pressing the right arrow on the bar with the sheet names. You will now be using the next 4 sheets to work on z* analysis.

1. **z star plate map**: This map is for your reference so you can visualize how the experiment was set up for z* analysis. As discussed above, the controls look different for this plate since our samples act as their own controls. So we only have ALT- control cells and they are scattered across the plate rather than clustered in one column to help check for experimental error.
2. **z star raw data telomeric plate**: This is what qPCR data looks like when taken directly from the software. It is set up the same way as the raw data readout for the Z’ plate, but you will notice the samples are arranged differently because the experimental plate map is not the same for z* analysis. This sheet provides the Ct values for the telomeric DNA plate of the z* experiment.
3. **z star data RPLP0 plate**: This sheet provides the Ct values for the RPLP0 plate of the samples in the z* experiment.
4. **z star analysis**: This is where you will calculate the z* score for each sample.

Now it is time to begin your analysis on the **z star analysis sheet**.

The z* and MAD equations have been copied below:


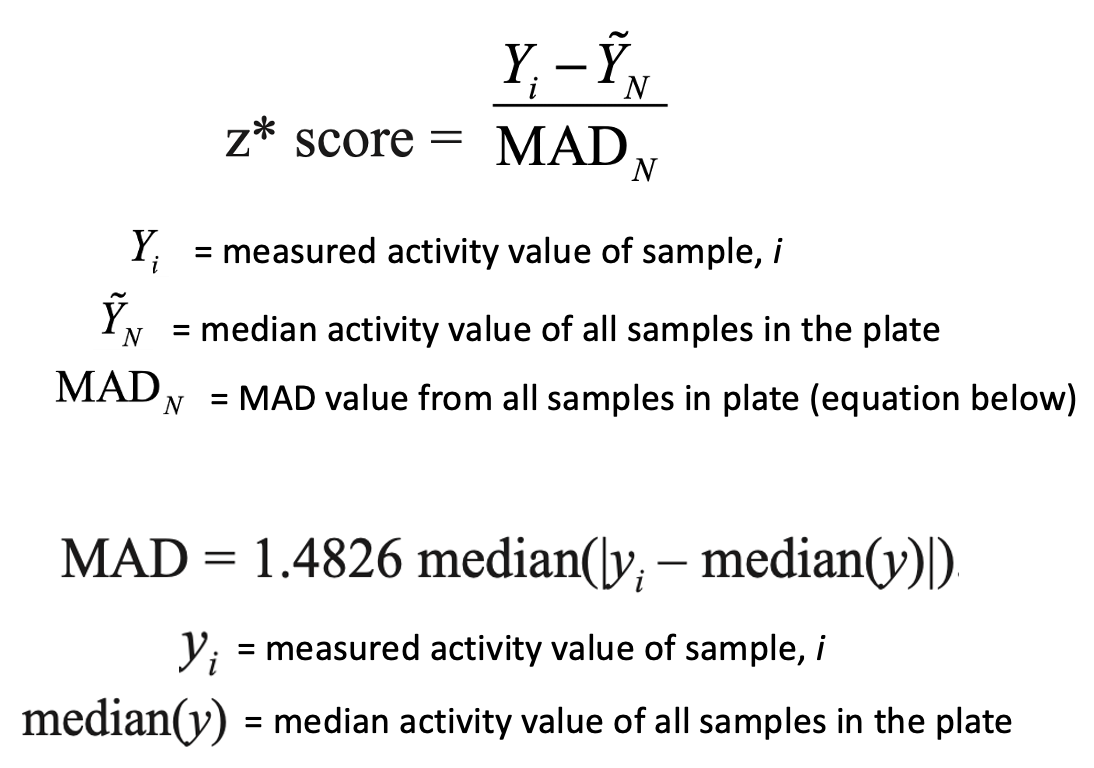


1. The telomeric DNA and RPLP0 Ct values for each sample are listed in the properly labeled columns. First, you need to calculate the delta Ct for each sample. You can do this using the same google sheets formulas used to calculate the delta Ct values for the Z’ plate.
2. Once you have a delta Ct value for each sample, you need to find the median delta Ct value for all samples. Do this by selecting cell G7 and typing “=median”. Select the median equation from the dropdown menu and then select cell D2 and drag your cursor all the way down the column to select all delta Ct values. This should give you the median delta Ct of all samples in the plate.
3. Next, you need to calculate the components of the MAD equation which is not only a component of the z* equation but also acts as our cutoff for determining hits. The MAD is calculated by first subtracting the median delta Ct from each individual delta Ct value. Then, the median of those values is calculated and multiplied by the number 1.4826, which corrects our data for not being normally distributed. The MAD value essentially acts as the median-based metric version of standard deviation. To calculate the MAD you first need to calculate the absolute value of each sample’s delta Ct minus the median delta Ct value of the dataset. Do this by selecting cell E2 and typing “=abs”. Select the absolute value equation from the dropdown, then select cell D2, type “-”, select cell G7, type a “$” symbol between the G and the 7, then type “)” to complete the equation. The full equation should read “=ABS(D2-G$7)”. This should give you the absolute value of the first sample’s delta Ct minus the median delta Ct.
4. Select cell E2 again and hover over the bottom right corner until the black cross appears. Click the corner with the cross cursor and drag it down the column until you have selected all of the cells in column E that correspond to a sample in column A. This should calculate the absolute value of sample delta Ct minus median delta Ct for all samples. The dollar sign in the middle of G7 ensures that the median delta Ct value stays constant in the equation while the sample delta Ct changes as you go down the column.
5. Now you need to find the median of the values listed in column E. Select cell G10 and type “=median”. Select the median formula from the dropdown menu and select all the values listed in column E. This should give you the median of the absolute value of sample delta Ct minus median delta Ct.
6. Now you can calculate the MAD value. Select cell G13 and type “=1.4826*” and select cell G10. This should give you the MAD value, which you will use in the z* equation. For detecting hits, we use the cutoff of plus or minus 3 * MAD (or 3MAD) so we will need to calculate 3MAD as well. Do this by selecting cell H13 and typing “=3*” and select cell G13. This will calculate 3MAD which you will use when you plot the z* scores.
7. Next, you will need to calculate the z* score for each sample. Select cell F2, type “=(“, select cell D2, type “-”, select cell G7, type a “$” between G and 7, and type “)/”. Then complete the equation by selecting cell G13 and typing “$” between G and 13. The full equation should read “=(D2-G$7)/G$13)”. This will give you the z* score for the first sample listed.
8. To get the z* score for all samples, select cell F2 and hover over the bottom right corner until the black cross appears. Click the black cross and drag it down until you have selected all the cells in the column associated with a sample in column A. This should calculate the z* score for each sample and the “$” character should have kept the median delta Ct and MAD values constant throughout the entire column.
9. The next step is plotting the z* scores in a scatter plot. Do this by clicking “insert” the the tool bar and selecting “chart” from the dropdown menu. An empty chart and a chart editor menu should pop up (see image below)


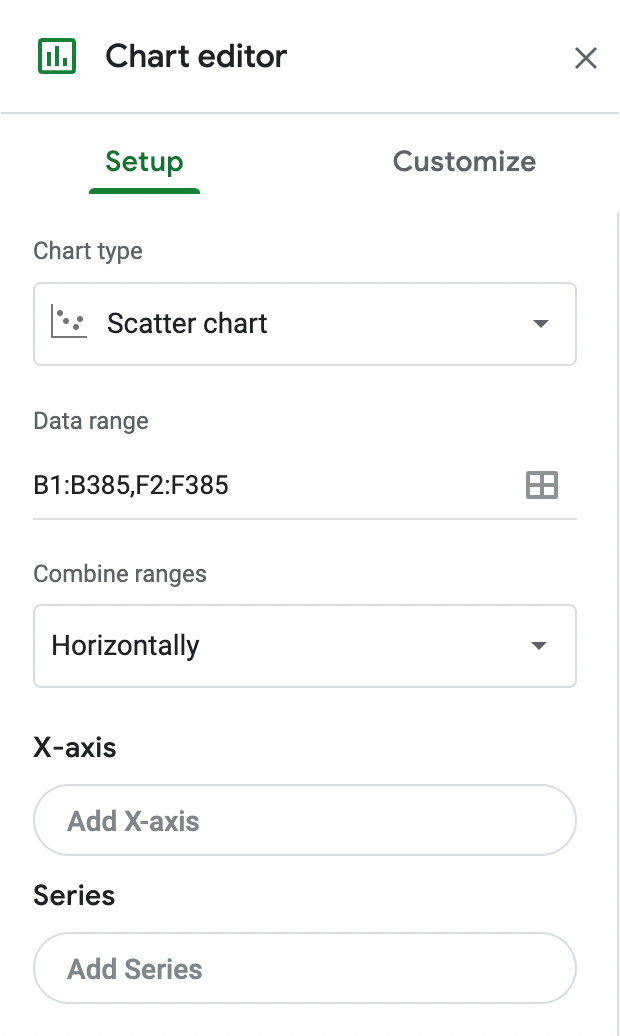


1. Click the box under “chart type” and select “scatter plot” from the dropdown menu. Below chart type the next component should say “data range” and there should be a box made up of four squares to the right of the section. If you hover your cursor over this box a prompt should come up that says “select data range”. Click this box and delete whatever is in the box right below the “select data range” header. Click the first z* value in the z* score column then drag down until you’ve selected all z* scores. This should fill in the data range box in the pop up. Once the proper data range has been selected click ok. Before continuing be sure that the box in the chart editor labeled X-axis remains empty. If this has a data range in it be sure to remove it as it will interfere with how the plot looks later on.
2. You should then see all your z* scores plotted out in a scatter plot. In order to identify hits, you need to look for samples with z* scores that fall above or below the 3MAD value. To do this you need to plot 2 lines, one at y=+3MAD and one at y=-3MAD. To do this, select cell I2 and type in your positive 3MAD value. Copy paste this value all the way down the column until you reach the last sample labeled in column A. You should end up with a column made up of the same number repeated which will give you a line at y= 3MAD on your chart.
3. Do the same in column J but instead of repeating the positive 3MAD value, list the negative 3MAD value.
4. Now, click on your chart so that the chart editor pops up and select “add series”, which should be listed below the header labeled “series”. When you click “add series” there should be a drop down menu with another box made up of four squares in the right of the dropdown menu. When you click this box a small window should pop up that says “select data range”. Click the entry box and then select the first cell in column I with the 3MAD value then drag down so that you have selected all the values in the column. Once you click ok, a trendline at the 3MAD value should appear on your chart. Do the same steps to add a series that represents the negative 3MAD trendline.

**Z* Calculations**

| Median |  |
| --- | --- |
| MAD |  |

Where are the negative control samples on the your graph? Is this where you expect a sample with no C-circles to be? Explain in 1-2 sentences why or why not.

Are there any compound treated samples that you would identify as hits that inhibit ALT activity? Explain how you determined that these compounds are causing a significant change in C-circle level.

3. Rate your level of confidence using the scale below to:

LO1: Define phenotypic cell-based screening & identify appropriate screening controls

1. Very Confident - I can do this easily
2. Confident - I may need to check resources but could figure it out
3. Somewhat Confident- I may need to ask for help but could figure it out
4. Poorly Confident - Even with resources and help it would still be vague
5. Not Confident - I can’t do it

LO2: Apply statistical modeling to a phenotypic screen to identify biologically meaningful results

1. Very Confident - I can do this easily
2. Confident - I may need to check resources but could figure it out
3. Somewhat Confident- I may need to ask for help but could figure it out
4. Poorly Confident - Even with resources and help it would still be vague
5. Not Confident - I can’t do it

LO3: Interpret the biological significance of a z-value.

1. Very Confident - I can do this easily
2. Confident - I may need to check resources but could figure it out
3. Somewhat Confident- I may need to ask for help but could figure it out
4. Poorly Confident - Even with resources and help it would still be vague
5. Not Confident - I can’t do it
